# Meniscal Transplant surgery or Optimised Rehabilitation full randomised trial (MeTeOR2): a study protocol

**DOI:** 10.1136/bmjopen-2024-085125

**Published:** 2024-06-03

**Authors:** Susanne Arnold, Timothy Spalding, Helen Parsons, David Beard, Helen Bradley, Peter Crisford, David R Ellard, Manuela Ferreira, Alan Getgood, Jonathan Guck, Aminul Haque, Iftekhar Khan, James Mason, Bryony Milroy, P Myers, David Parker, Andrew James Price, Amy Smith, NA Smith, Toby Smith, Kimberley Stewart, Martin Underwood, Peter Verdonk, Andrew Metcalfe

**Affiliations:** 1 Warwick Clinical Trials Unit, Warwick Medical School, University of Warwick, Coventry, UK; 2 Cleveland Clinic London, London, UK; 3 University Hospitals Coventry and Warwickshire, Coventry, UK; 4 University of Oxford, Oxford, UK; 5 Patient Representative, Coventry, UK; 6 University of Sydney Institute of Bone and Joint Research, Saint Leonards, New South Wales, Australia; 7 Western University, London, Ontario, Canada; 8 Brisbane Orthopaedic & Sports Medicine Centre, Brisbane, Queensland, Australia; 9 Faculty of Medicine and Health, The University of Sydney, Sydney, New South Wales, Australia; 10 University of Antwerp, Antwerpen, Belgium

**Keywords:** Knee, Randomized Controlled Trial, REHABILITATION MEDICINE, SURGERY, TRANSPLANT SURGERY

## Abstract

**Introduction:**

Pain and disability after meniscectomy can be a substantial lifelong problem. There are few treatment options, especially for young people. Non-surgical management (rehabilitation) is an option but increasingly surgeons are performing meniscal allograft transplants (MATs) for these individuals. However, this is still an uncommon procedure, and availability and usage of MAT vary widely both in the UK and internationally. It is not known which treatment option is the most effective and cost-effective.

**Methods and analysis:**

The Meniscal Transplant surgery or Optimised Rehabilitation trial is an international, multicentre, randomised controlled trial. The aim is to compare the clinical and cost effectiveness of MAT versus an optimised package of individualised, progressive, rehabilitation that we have called personalised knee therapy (PKT).

Participants will be recruited from sites across the UK, Australia, Canada and Belgium. The planned 144 participants provide at least 90% power to detect a 10-point difference in the Knee injury and Osteoarthritis Outcome Score (KOOS4) at 24-months post randomisation (primary outcome). A prospectively planned economic evaluation will be conducted from a healthcare system and personal social services perspective. Secondary outcome data including health utility, occupational status, sports participation, mental well-being, further treatment, and adverse events will be collected at 3, 6, 12, 18, and 24 months. Analysis will be on an intention-to-treat basis and reported in-line with the Consolidated Standards of Reporting Trials statement.

**Ethics and dissemination:**

The trial was approved by the London—Bloomsbury Research Ethics Committee on 19 August 2022 (22/LO/0327) and Northern Sydney Local Health District Human Research Ethics Committee, NSW, Australia on the 13 March 2023 (2022/ETH01890).

Trial results will be disseminated via peer-reviewed publications, presentations at international conferences, in lay summaries and using social media as appropriate.

This protocol adheres to the recommended Standard Protocol Items: Recommendations for Interventional Trials (SPIRIT) checklist.

**Trial registration number:**

ISRCTN87336549.

**STRENGTHS AND LIMITATIONS OF THIS STUDY**

- International, multicentre, randomised controlled trial comparing an initial treatment strategy of meniscal allograft transplant (MAT) or personalised knee therapy (PKT) for adults with post-meniscectomy pain and functional loss.
- Participants will be recruited from sites across the UK, Australia, Canada and Belgium.
- Clinical outcomes assessed using the Knee Injury and Osteoarthritis Outcome Score in addition to a range of secondary outcomes.
- Rare clinical presentation that may result in difficulty recruiting.
- No radiological or biochemical measures to determine if MAT changes risk of osteoarthritis.

## Introduction

A meniscectomy is an arthroscopic procedure where part or all of the meniscus is removed following an irreparable tear. Meniscal tears are common, often as a result of sporting injuries in young people.^1^

Every year, approximately 80 000 people in England undergo meniscectomy.^2^ For the majority of people, pain and other symptoms, such as locking, improve after surgery. However, some people have ongoing problems with pain and functional loss, leading to many years of disability. Ten years after a meniscectomy, 20% of people have developed osteoarthritis, increasing to 50% after 20 years.^3 4^ An individual aged 30–39 years having a partial meniscectomy is 40 times more likely to need a knee replacement after 15 years than that of the general population.^5^

There are few treatment options for people suffering from post-meniscectomy problems. Knee replacement is not usually recommended until people are over 50 years old. Therefore, younger adults with post-meniscectomy pain often have to live with their symptoms until they are at an age when knee replacement may be considered appropriate.^6 7^ Increasingly, orthopaedic surgeons are performing meniscal allograft transplants (MATs) for people with post-meniscectomy pain.^8^ Availability, access and usage of MAT are, however, variable across the clinical community, resulting in a potential inequity in access to treatment. The transplant, which involves taking a donor meniscus from someone who has died and inserting it into someone with a similar-sized knee, is costly, at around £7500 per case in the UK, although costs will vary substantially worldwide.

Non-surgical management (optimised rehabilitation) is also a viable treatment option.^9^ The principles of care for early osteoarthritis could apply to this population, as rehabilitation has a strong evidence base in this (related) setting. Potential treatments include exercise, weight loss, lifestyle and activity advice, adjuncts such as orthoses and offloading braces, and non-steroidal anti-inflammatory drugs (NSAIDs); it is reasonable to think they could have a similar beneficial effect in the post-meniscectomy pain setting.^10–13^ If shown to be effective for persistent pain after meniscectomy, a package of optimised rehabilitation would be safer, and cheaper, and does not impact on an individual’s ability to work or undertake other activities compared with a postoperative phase.

There is a lack of high-quality evidence about whether MAT or non-surgical care is more effective and which is the most cost-effective treatment for post-meniscectomy pain and functional loss. Our 2019 systematic review included 19 studies (n=1731); 18 case series of MAT and just one randomised controlled trial (RCT) (this being a pilot study, n=21).^4^ Patient-reported outcomes improved from baseline across all studies. There are no data at present on non-surgical care for people with pain after meniscectomy; it would be very hard to make many meaningful comparisons of a programme unless baseline differences between groups were balanced through randomisation.

Currently, there are no comparative data (except our previous pilot study)^14^ to inform patients, clinicians or health commissioners about whether MAT should be used or whether patients with post-meniscectomy pain would be better treated with non-surgical interventions.^4^

## Aim

The aim of the study is to compare the clinical and cost-effectiveness of an initial treatment strategy of MAT or Personalised Knee Therapy (an optimised package of rehabilitation) for adults with post-meniscectomy pain and functional loss.

## Methods and analysis

### Trial design

The Meniscal Transplant surgery or Optimised Rehabilitation (MeTeOR2) trial is an international, multicentre, two-arm RCT comparing the clinical and cost- effectiveness of MAT with an optimised package of rehabilitation termed PKT. The planned trial start date was June 2022. The planned trial end date is November 2027.

**Figure 1:**
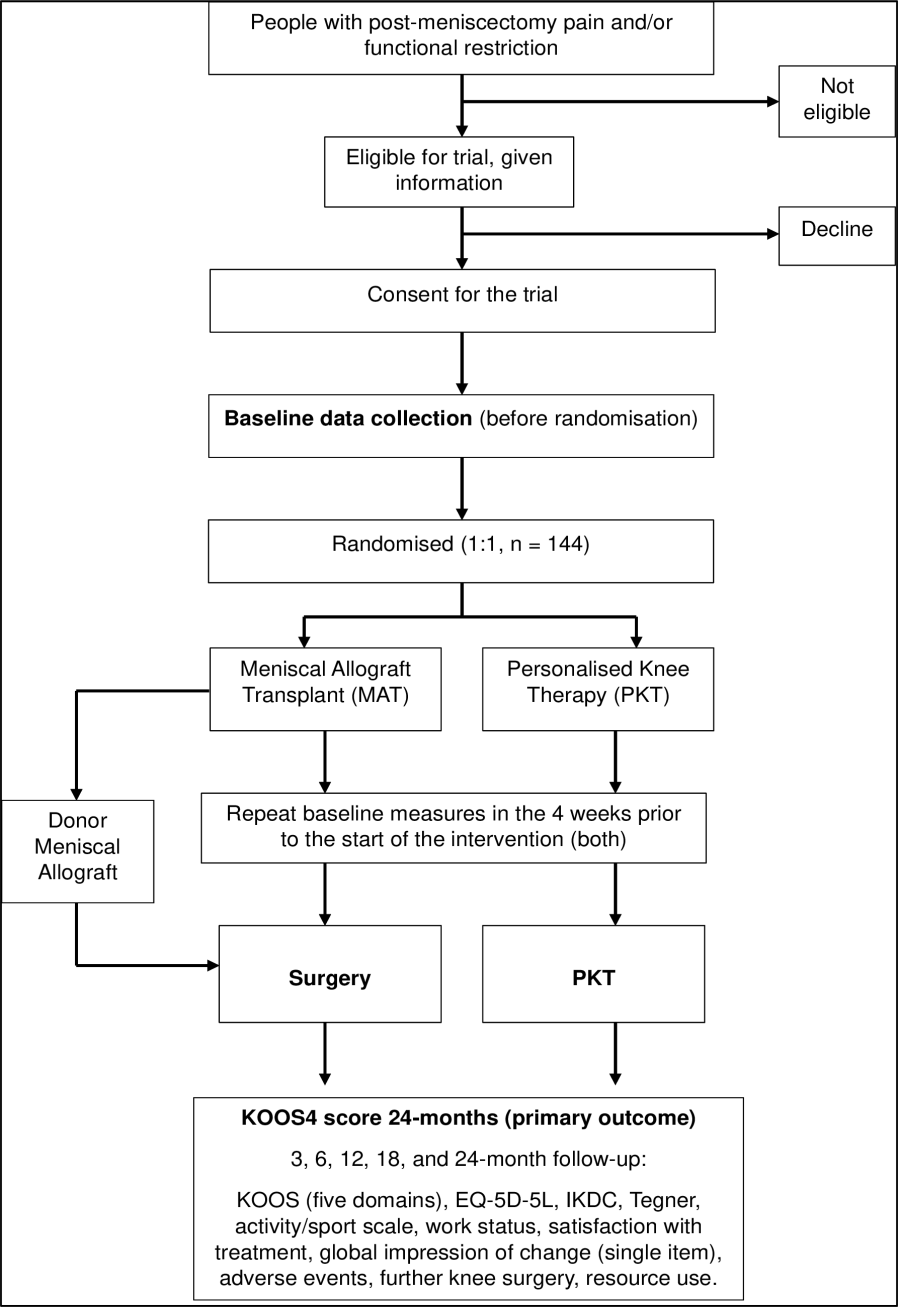
Meniscal Transplant surgery or Optimised Rehabilitation (MeTeOR2) participant flow diagram. IKDC, International Knee Documentation Committee; KOOS4, Knee injury and Osteoarthritis Outcome Score; EuroQol five-dimension questionnaire and Euroqol visual analogue scale (EQ-5D-5L).

This paper and the trial protocol were written following the Standard Protocol Items: Recommendations for Interventional Trials (SPIRIT) guidelines. Figure 1 shows the participant flow diagram, and a copy of the participant consent form is included in online supplemental file 1. A summary of core trial information is presented in the WHO trial registration data set in online supplemental file 2.

10.1136/bmjopen-2024-085125.supp1 Supplementary data

10.1136/bmjopen-2024-085125.supp2 Supplementary data

### Patient and public involvement (PPI)

Patient involvement has been critical in the design and development of the trial and will continue to be important in its delivery, including the interpretation and dissemination of the results. As well as running several PPI events with patients who have experience of post-meniscectomy pain and either PKT or MAT, our research team includes two PPI representatives as coapplicants. Our PPI coapplicants are integral to the Trial Management Group (TMG) and are engaged in trial management meetings, contributing the patient perspective to trial processes and procedures and will be key to our dissemination plan. Two further people with post-meniscectomy pain sit on the Trial Steering Committee (TSC).

### Objectives

#### Primary objectives

- To compare the clinical effectiveness of an initial treatment strategy of MAT compared with PKT, based on participant-reported knee function at 24-months post-randomisation, using the four-domain version of the Knee Injury and Osteoarthritis Outcome Score (KOOS4).
- To determine the cost effectiveness of MAT compared with PKT from a National Health Service (NHS) and Personal, Social Service (PSS) perspective.

#### Secondary objectives

- To quantify and draw inferences on health utility, occupational status, sports participation, mental well-being, further treatment (including further surgery or physiotherapy in either arm) and adverse events (AEs) at 3, 6, 6, 12, 18 and 24-months.
- To evaluate process measures to compare days to initiation of treatment, rehabilitation attendance, and participant expectation of outcome.

### Outcome measures

Outcome measures were chosen in collaboration with our PPI representatives and our experienced clinical team. In line with SPIRIT guidance, details of the schedule of enrolment, interventions, and assessment can be found in online supplemental file 3.

10.1136/bmjopen-2024-085125.supp3 Supplementary data

#### Primary outcome

The primary clinical effectiveness outcome is participant-reported knee function using the four-domain KOOS4 score 24-months after randomisation.^15^ This is a knee-specific instrument (0–100, 100 best score) incorporating four of the five domains of the full KOOS score (KOOS4 uses the domains for symptoms, pain, function/sports and quality of life, but not activities of daily living).^15^ The KOOS4 has been widely used in previous trials of knee surgery including those with young adult, non-arthritic populations and is well accepted by the clinical community.^14 16–19^

Cost effectiveness of the interventions will be assessed using health utility, occupational status, sports participation, mental well-being, further surgery (treatment switching or secondary knee surgery), satisfaction with the outcome of treatment, participant global impression of change and AEs.

#### Secondary outcomes

All collected at 6, 12, 18 and 24 months. As an additional pre-intervention baseline measure (to understand any changes occurring whilst participants wait for their intervetnion, given the potetnial discrepancy between groups), we will collect KOOS4, EQ-5D-5L, IKDC, Tegner and SWEMWBS scores in the four weeks leading up to the start of the participant's intervention.

- The KOOS4 (also recorded at pre-intervention).
- The five individual KOOS domains (also recorded at baseline). A validated knee-specific instrument developed to assess the patients’ opinion about their knee and associated problems.
- Health utility using EQ-5D-5L (also collected at baseline, preintervention and 3 months).^20 21^
- Short Warwick-Edinburgh Mental Wellbeing Scale (SWEMWBS, also collected at baseline and pre-intervention).^22 23^
- Tegner activity/sport scale (also collected at baseline and pre-intervention).^24^
- Satisfaction with the outcome of treatment using a 5-point Likert scale.^25^
- The Participant Global Impression of Change. A single-item, 7-point Likert scale.^26^
- The International Knee Documentation Committee (IKDC, also collected at baseline, pre-intervention and 24 months).^27^
- Further knee surgery and physiotherapy.
- Resource use using participant questionnaires (also collected at 3 months).
- Analgesia use.

#### Safety outcomes

- AEs and serious adverse events (SAEs) related to the surgery, anaesthetic, or rehabilitation will be collected according to relevant Warwick Clinical Trials Unit (WCTU) Standard Operating procedures (SOPs) at 3, 6, 12, 18 and 24 months post randomisation.

#### Process and fidelity measures

- Days to initiation of randomised treatment, defined as the number of days between randomisation and the first physiotherapy contact for those who undergo PKT or the day of surgery.
- Physiotherapy attendance and intervention details will be recorded on physiotherapy case report forms (CRFs) at each appointment.
- A surgical CRF will be completed after each surgery including details about the anaesthetic and surgery such as surgical findings, surgical time, tourniquet time, graft size, fixation of graft, and additional procedures.

Alongside a detailed data management plan, the programming team at WCTU have built a bespoke database management system to store and maintain high-quality data for the duration of the trial.

### Eligibility criteria

#### Inclusion criteria

- Pain and/or functional restriction from the knee, severe enough to warrant potential MAT in the judgement of the treating clinician.
- Previous meniscectomy ≥6 months ago.

#### Exclusion criteria

- Symptomatic ligament instability, not previously corrected, as determined by the assessing clinician
- Coronal limb alignment which requires surgical correction (previous correction, performed at least 6 months before entry to the trial, is not an exclusion criteria), as determined by the assessing clinician (Previous surgery (except prior MAT) will be allowed. Where people have mal-alignment or ligament deficiency (30% of the MAT population), alignment or ligament deficiency may be corrected by osteotomy or ligament reconstruction, which should have been performed at least 6 months before entering someone into the study).
- Age under 16 years, or if ≥16 years, open growth plate at the proximal tibia as judged by the clinical team on imaging taken as part of standard care.
- Full-thickness cartilage loss (exposed bone) >1 cm^2^ on routine clinical MRI, prior surgery, or any other form of clinical imaging or evaluation. This will be determined by the assessing clinician (it could be based on an assessment by a clinician or a radiologist, although the final decision rests with the treating clinician).
- Inflammatory arthritis affecting the study knee as determined by the assessing clinician (ie, a prior inflammatory event not considered to be related to the current clinical condition would not require exclusion).
- Unable or unwilling to engage with rehabilitation.
- Unable to adhere to trial processes.
- Previous randomisation in the present trial (ie, other knee). Where a previous randomisation has occurred in error, a participant may be withdrawn and this criterion will not apply.

### Participant identification, screening and withdrawals

Potential participants will be identified by clinical teams across the UK, Australia, Canada, and Belgium. Currently, sites in the UK and Australia are recruiting participants (UK ethical approval obtained 22 August 2022 and open to recruitment 7 March 2023, Australian ethical approval obtained 4 April 2023 and open to recruitment 8 December 2023). The centre in Canada has obtained ethical approval (20/11/2023) and plans to open to recruitment in Summer 2024. Ethical approval for sites in Belgium is pending but without a confirmed timeline yet.

Participant identification will typically happen in outpatient or intermediate care clinics, hospital waiting lists, patient discharge lists or referrals into hospital (from either primary, intermediate, or secondary care). Any potential participants will be assessed for eligibility by an appropriately delegated clinician. There is no requirement for any specific investigation although it is normal clinical practice to evaluate someone with a painful knee after prior meniscectomy with MRI scan for diagnostic purposes before any consideration of treatment options. This is likely to be the case for most participants but is not a requirement for entry. A screening log will be completed directly onto the trial database at all sites.

Those who are eligible will be given information about the trial and invited to discuss it further with a member of the local research team. They will be given adequate time to consider participation. Information sheets, invitation letters and other approved patient-facing materials may be posted, emailed, physically provided or shared via other means to potential participants. A member of the local research team will carry out the informed consent process, collecting either written consent or witnessed remote verbal consent, and baseline data collection.

### Randomisation

After consent and baseline data have been collected, participants will be randomly allocated to the two treatment groups via a central computer-based randomisation system provided by WCTU, independent of the study team. Randomisation will be a 1:1 allocation determined by a minimisation algorithm using age (≥30 years or <30 years), treating site and knee compartment (lateral or medial) as factors, with a random factor included to provide a 70% weighting towards balance across the trial.

Participants will be randomised sequentially at site level. Randomisation will be performed by any member of the local clinical or research team on the delegation log, using the online system. Depending on local site arrangements, stickers, electronic tags or an equivalent may be used on the participant’s clinical notes to flag their inclusion in the trial.

Randomised participants can choose to discontinue their treatment and/or withdraw from follow-up at any time, without prejudice. This will have no effect on their current or future care. All withdrawals will be monitored by the TMG and oversight committees.

### Trial treatments(s)/interventions

#### Group 1: MAT (surgical intervention)

Participants allocated to surgery will receive MAT once an allograft becomes available. Surgical procedures are outlined in a trial-specific surgical manual which was developed at a surgical consensus meeting (July 2022) and informed by the International Meniscus Reconstruction Experts Forum (IMReF) guidelines.^8^

Immunosuppression is not required as meniscal allografts have low cellular content and are not rejected in the way solid organ transplants might be. Donors are screened for blood-borne diseases according to approved tissue bank policies. Essentially, this is similar to blood donors and there are no reported cases of viral transmission from MAT.

It is possible that participants may have to wait for 6–12 months for a graft of the right size to become available (median 6.5 months in our pilot study).^14^ The delay to receive a graft is unavoidable in meniscal transplantation surgery, due to the availability of a suitable-sized donor graft from tissue banks. To reduce time to surgery, the dimensions of the graft needed will be sent to tissue banks according to surgeon preference and usual practice. Recovery following MAT plateaus after 9–12 months post surgery. As the primary outcome is at 24 months, recovery will still be completed by the primary outcome time point, even for people in whom surgery is delayed.

All care, including the choice of anaesthetic, the surgical procedure and post-operative analgesia, will be in accordance with usual procedures and care at participating sites.

Rehabilitation for the surgery group will be according to a standardised programme specific to MAT. We have used the lead centre’s established programme for this and, in discussion with participating centres, adapted it to ensure that it is deliverable across multiple NHS and international sites. A formal PKT programme will not be used prior to surgery in the MAT arm, although we do not discount people having prior or current physiotherapy.

#### Group 2: personalised knee therapy (non-surgical intervention)

The PKT programme is a tailored and optimised package of rehabilitation aimed at improving individualised outcomes for people with knee pain and/or functional limitation following meniscectomy. It was developed from a pilot study,^14^ and refined through literature and guideline reviews on non-surgical interventions including physiotherapy and other conservative interventions such as weight loss advice, knee braces and orthotics and referral to other services.^9–13 28–30^ This was supported with a consensus meeting held in July 2022, whereby experts in the field came together to develop a final comprehensive evidence-based PKT programme for the trial, ensuring that it is deliverable across all of our UK and international study sites. A template for intervention description and replication (TIDieR) checklist^31^ for describing interventions for the METEOR2 PKT intervention is included as online supplemental file 4.

10.1136/bmjopen-2024-085125.supp4 Supplementary data

The PKT programme is outlined below:

##### Aim

To reduce pain, restore full knee range of motion, improve knee function and optimise overall social participation through a goal-setting approach personalised to the participant.

##### Delivered by

Physiotherapists trained in the principles of the METEOR2 PKT programme. Training includes the background to the problem of post-meniscectomy pain, the rationale for the trial, discussion about the PKT framework and trial administration procedures. Trained physiotherapists receive a comprehensive PKT manual containing a detailed account of all trial and intervention procedures.

##### Mode of delivery

As the intervention will be personalised to the participant, there is flexibility, as determined by clinical judgement and service provision, for PKT to be delivered face-to-face in acute hospital or community physiotherapy departments, through virtual consultation or a hybrid of the two.

##### Duration

Minimum of 3 months from the first assessment and a minimum of 4 sessions in total, but can be as many as clinically required, reflecting normal clinical practice.

##### Treatment starting point from randomisation

When an appointment with an appropriate physiotherapist is available, according to normal clinical waiting times.

##### Timing of consultations

The interval between consultations will be personalised to the needs of the participant based on their presentation, progress and treatment goals.

##### Assessment

Initial assessment will include participant’s history (subjective assessment) and physical examination (objective assessment). This will follow a routine musculoskeletal physiotherapy assessment. Specific focus in the objective assessment will be made on lower limb function and kinetic control, muscle length, strength, and recruitment. Shared goals will be discussed between physiotherapist and participant which will form the basis of the problem list and treatment plan.

##### Treatments

Based on the individualised problem list and goals developed by the physiotherapist and participant, the physiotherapist will deliver interventions aimed to specifically manage the presenting problems. Through this, a personalised approach is made to the participants rehabilitation, optimising their outcome. Each participant will be provided with a manual which will include their treatment goals, information about their PKT programme with details of specific individualised exercises, and an exercise planner to encourage adherence.

### End of trial

The trial will end when all 24-month follow-up data have been received and entered onto the trial database. The trial will only be stopped prior to this if mandated by the Research Ethics Committee (REC), following recommendation from the TSC, or if funding for the trial ceases.

The trial will be extended if we receive funding for 5, 10 or 15–20 year follow-up. Consent will be obtained for long-term follow-up.

## Safety reporting, AEs and SAEs

All AEs and SAEs will be defined using widely accepted standard criteria. For this trial, AEs and SAEs will be collected from the point of randomisation onwards, up to 24 months. To avoid unnecessary reporting, some events which occur during treatment and recovery will be considered as normal aspects of the therapy, anaesthetic and post-operative recovery processes unless in the opinion of the clinical team, they are untoward, excessive or outside of what might normally be expected for the procedure; these will not need reporting. We will only collect AEs and SAEs related to the participants’ knee, treatment they receive in the trial (or any treatment for the study knee), or trial processes.

SAEs will be reported to WCTU within 24 hours of the research staff becoming aware of the event. Events will be followed up until they are resolved, or until the end of the trial, and an outcome has been agreed.

## Statistical analysis

### Power and sample size

The target difference for KOOS4, widely used across multiple RCTs, is 10 points (on a 0–100 scale). This is consistent with anchor-based studies and is accepted as a clinically meaningful difference.^16–18 32^ This effect is similar to that found for knee cartilage repair on autologous chondrocyte implantation.^17 33^ Cartilage repair was found to be clinically and cost-effective and it was subsequently recommended by the UK’s National Institute for Health and Care Excellence (NICE).^34^ MAT is about half the cost, so if it has a similar benefit it should be cost-effective.^33^

The pooled SD in our pilot study was 14.5.^14^ Allowing for the multicentre nature of this trial, along with the small size of the pilot, we have used the upper boundary of the 60% CI to estimate the SD. According to the method of Chen *et al*,^35^ this was 16.4. In this competency-based trial, each site will contribute small numbers and adjustment for clustering is not necessary. Hence, for a two-group parallel arm design, assuming 90% power and two-sided 5% significance, we would require a sample size of 116. Allowing for 20% loss to follow-up, the required sample size is 144.

### Statistical analysis plan

All analyses will be reported in accordance with Consolidated Standards of Reporting Trials guidelines.^36 37^ A comprehensive statistical analysis plan will be agreed with the Data Monitoring Committee (DMC) prior to any formal analysis. Descriptive statistics will be constructed for baseline data to check for any characteristic differences between allocation groups.

A generalised linear model will be used to assess differences in the KOOS4 at 24-months post-randomisation. As a minimum, adjustment terms for allocation, age and baseline score will be used. Variables found to be unbalanced at baseline may also be fitted, if judged appropriate. Where possible, a random effect for centre or country effects will also be used. Secondary outcomes will be analysed using a similar approach to the primary analysis where data type and distribution allow. Outcomes which are categorical in nature (eg, patient global impression of change) will be analysed using proportional linear or logistic regression and subject to the same variable adjustments. To assess the effects of treatment switching, we will construct models to compare participants on an ’as treated‘ basis. That is, compare outcomes for those who received each treatment, regardless of allocated group. Exploratory models will be performed to assess the change from pre-intervention scores to the 24-month outcome. This may include assessing the trajectory of recovery over time using latent growth models, or assessing variables as prognostic or mediating factors. Pre-specified subgroup analyses will be performed for affected compartment (medial or lateral), age (30 years or over/under 30 years) and gender. Where possible, reasons for missing data will be ascertained and reported. If judged appropriate, effects of missing data will be explored using multiple imputation.

#### Database

All data will be stored in a customised database system developed by the experienced programming team at WCTU. This is supported by a detailed data management plan produced in accordance with WCTU SOPs to ensure high-quality data collection over the duration of the trial.

## Health economic evaluation

A prospectively planned economic evaluation will be conducted from a healthcare system and personal social services perspective, according to the recommendations of the NICE reference case.^38^ Given multi-national recruitment, we will consider inter-country healthcare differences in constructing our analysis plan.^38 39^

Health service contacts, made in connection with their treatments, will be recorded as part of the resource utilisation questionnaires at 3, 6, 12, 18 and 24 months. Time lost from work (paid/unpaid) will also be recorded. Participants will be encouraged to use an electronic or paper calendar to help recall this information at follow-up. Intervention and sequelae healthcare resource use will be costed using most recently available UK-published national reference costs, reflated to a common year.^40 41^

EQ-5D-5L scores will be converted to health status scores using the UK value set recommended by NICE guidance at the time of analysis.^42^ Using the trapezoidal rule, the area-under-the-curve of health status scores will be calculated, providing patient-level quality of life year (QALY) estimates. QALYs will be estimated for the whole cohort, applying UK values.

If the level of missingness data is below 5%, complete case analysis will be conducted. If not, mechanisms of missingness of data will be explored and multiple imputation methods will be applied to impute missing data. Complete case data or imputation sets will be used in bivariate analysis of costs and QALYs to generate incremental cost per QALY estimates and CIs.^43–46^ Findings will be analysed and visualised as cost-effectiveness acceptability curves, net monetary benefit and value of information analysis. The potential for heterogeneity of cost-effectiveness findings by country will be explored by fitting interaction terms to models, and if necessary performing country-specific analysis applying local costs to the complete clinical effectiveness data. A UK cohort-only analysis will be included within planned secondary analyses. If the pattern of costs and benefits is non-convergent or non-dominant at 24 months, we will develop a decision analytic model, using our expertise in economic modelling in this field.^4 33 47^

## Ethics and dissemination

The trial was approved by the London—Bloomsbury REC on 19 August 2022 (22/LO/0327) and Northern Sydney Local Health District Human REC, NSW, Australia on 13 March 2023 (2022/ETH01890). The trial will adhere to the Declaration of Helsinki and Good Clinical Practice, following all relevant WCTU SOPs. Participants will provide informed consent before agreeing to participate. An independent DMC and TSC will provide oversight from set-up to the closure of the trial. Using the definition provided by NIHR and WCTU SOPs, both committees will comprise independent members who will sign a separate committee charter. Data monitoring plans will be implemented by the trial sponsor. All protocol amendments will be communicated to sites by the trial coordinating team.

### Data sharing

De-identified data that underlie the trial results will be available for non-commercial use, up to 1 year after publication of the primary outcome trial findings, or from metadata stored in a University repository up to 10 years without investigator support. Third party access to trial data must be via a data-sharing agreement with the sponsors. They must have an ethically approved protocol in place for use of the data, and agree the approved protocol with the MeTeOR2 TMG. Data may be used for commercial purposes, according to the conditions above, but will need specific agreements in place prior to access being agreed; this may include a license fee. Analyses may include individual patient data meta-analyses or other purposes as agreed with the MeTeOR2 TMG.

Available data will include (but is not exclusive to) deidentified individual participant data, the study protocol, Statistical Analysis Plan (SAP), informed consent sheets, and analytic codes used.

### Trial registration and study timelines

The trial is registered with the ISRCTN register (ISRCTN87336549). The current version of the protocol is V.2.0, approved on 24 November 2023. The planned dates of the study are from June 2022 to November 2027.

### Dissemination and publication

Trial results will be shared with trial collaborators initially, with the main results paper being drafted by the trial team and agreed by the TSC before submission to a major peer-reviewed publication. Results will be disseminated at international meetings and conferences. Presenting results to patients and the public will be led in conjunction with our patient partners. Dissemination to trial participants will follow current Health Research Authority guidelines, with summaries provided on the METEOR2 website and social media as appropriate.

## Supplementary Material

Reviewer comments

Author's manuscript
