# Supplementary material for: Meniscal Transplant surgery or Optimised Rehabilitation full randomised trial (MeTeOR2): a study protocol
Source: BMJ Open. 2024 Jun 3;14(6):e085125. doi: 10.1136/bmjopen-2024-085125 (PMC11149128; doi:10.1136/bmjopen-2024-085125)
Supplement: Supplementary data [file bmjopen-2024-085125supp002.pdf]

Supplementary File 2: METEOR2 WHO trial registration data set

Data Category	Information
Primary Registry and Trial Identifying Number	ISRCTN
Date of Registration in Primary Registry	02/09/2022
Secondary Identifying Numbers	NIHR HTA: 131629 , IRAS: 307686
Source(s) of Monetary or Material Support	National Institute for Health Research, Health Technology Assessment
Primary Sponsor	University of Warwick
Contact for Public Queries	meteor2@warwick.ac.uk
Contact for Scientific Queries	Mr Andrew Metcalfe (Co-CI), Warwick Clinical Trials Unit, University of Warwick.
Public Title	For people with pain after a meniscectomy, but without established OA, does a treatment strategy of undertaking MAT surgery or personalised knee therapy result in better clinical and/or cost effectiveness outcomes?
Scientific Title	The Meniscal Transplant Surgery or Optimised Rehabilitation - Full Randomised Controlled Trial
Countries of Recruitment	UK, Australia, Canada, Belgium.
Health Condition(s) or Problem(s) Studied	People with pain after meniscectomy but without established osteoarthritis
Intervention(s)	Surgical Intervention: Meniscal Allograft Transplant (MAT)<br>Non-surgical Intervention: Personalised Knee Therapy (PKT)
Key Inclusion and Exclusion Criteria	Inclusion criteria<br>i. Pain and/or functional restrictions from the knee, severe enough to warrant potential MAT in the judgement of the treating clinician.<br>ii. Previous meniscectomy more than 6 months ago.<br><br>Exclusion criteria:<br>i. Symptomatic ligament instability, not previously corrected, as determined by the assessing clinician.<br>ii. Coronal limb alignment which requires surgical correction, (previous correction, performed at least 6 months before entry to the trial, is not an exclusion criteria), as determined by the assessing clinician.<br>iii. Age under 16 years, or if ≥16, open growth plate at the proximal tibia as judged by the clinical team on imaging taken as part of standard care.<br>iv. Full thickness cartilage loss (exposed bone) >1 cm2 on routine clinical MRI, prior surgery, or any other form of clinical imaging or evaluation.

Data Category	Information
	v. Inflammatory arthritis affecting the study knee as determined by the assessing clinician.<br>vi. Unable or unwilling to engage with rehabilitation.<br>vii. Unable to adhere to trial processes.<br>viii. Previous randomisation in the present trial (i.e., other knee).
Study Type	Interventional<br>Allocation: randomised; individual assignment<br>Phase III
Date of First Enrolment	June 2023
Sample Size	144
Recruitment Status	Recruiting at time of submission
Primary Outcome(s)	1. Participant-reported knee function at 24 months, post randomisation, using the four-domain version of the Knee Injury and Osteoarthritis Outcome score (KOOS4).<br>2. To assess the cost effectiveness of MAT compared to PKT from an NHS and Personal, Social Service (PSS) perspective measured using health utility, occupational status, sports participant, mental wellbeing, further surgery (treatment switching or secondary knee surgery), satisfaction with the outcome of treatment, participant global impression of change and adverse events at three (EQ-5D 5L only), 6, 12, 18 and 24 months after randomisation
Key Secondary Outcomes	KOOS4, EQ-5D-5L, Short Warwick-Edinburgh Mental Wellbeing Scale, Tegner activity/sport scale, health resource use and analgesia use at baseline, pre-intervention, 6, 12, 18 and 24 months post randomisation.<br>International Knee Documentation Committee subjective score (IKDC) at baseline and 24 months post randomisation.<br>Satisfaction with the outcome of treatment, patient global impression of change, adverse events, further knee surgery at 6, 12, 18 and 24 months post randomisation.
Ethics Review	London – Bloomsbury Research Ethics Committee, August 2022.
