# Supplementary material for: Meniscal Transplant surgery or Optimised Rehabilitation full randomised trial (MeTeOR2): a study protocol
Source: BMJ Open. 2024 Jun 3;14(6):e085125. doi: 10.1136/bmjopen-2024-085125 (PMC11149128; doi:10.1136/bmjopen-2024-085125)
Supplement: Supplementary data [file bmjopen-2024-085125supp003.pdf]

Supplementary File 3. METEOR2 SPIRIT outcomes and assessment schedule

**Time point**	**-1**	**1**		**2**		**3**	**4**	**5**	**6**	**7**
Study period	Screening	Baseline	Pre-intervention	Intervention	Medical notes review	3-month	6-month	12-month	18-month	24-month
Check eligibility	✓									
Invitation to study	✓									
Informed consent		✓								
Medical history		✓								
Inclusion/exclusion criteria		✓								
Randomisation		✓								
Intervention (Surgery/PKT)				✓						
Operation note/Physio CRF				✓						
KOOS4		✓	✓				✓	✓	✓	✓
IKDC		✓	✓							✓
EQ-5D-5L		✓	✓			✓	✓	✓	✓	✓
Health resource use			✓			✓	✓	✓	✓	✓
SWEMWS		✓	✓				✓	✓	✓	✓
Tegner Activity Scale		✓	✓				✓	✓	✓	✓
Patient global impression of change							✓	✓	✓	✓
Complications, AEs, further surgery					✓	✓	✓	✓	✓	✓
Days to initiation of treatment				✓		✓	✓	✓	✓	
Rehabilitation attendance					✓	✓	✓	✓	✓	✓
