# Supplementary material for: Meniscal Transplant surgery or Optimised Rehabilitation full randomised trial (MeTeOR2): a study protocol
Source: BMJ Open. 2024 Jun 3;14(6):e085125. doi: 10.1136/bmjopen-2024-085125 (PMC11149128; doi:10.1136/bmjopen-2024-085125)
Supplement: Supplementary data [file bmjopen-2024-085125supp004.pdf]

Supplementary File 3: TIDieR Checklist for METEOR2 PKT Intervention

		Where located	
**Item number**	**Item**	**Primary paper (page number)**	**Other (details)**
**1**	**BRIEF NAME**<br>Provide the name or a phrase that describes the intervention	5, 14-15	N/A
**2**	**WHY**<br>Describe any rationale, theory, or goal of the elements essential to the intervention	14-15	N/A
**3**	**WHAT**<br>Materials: describe any physical or informational materials used in the intervention, including those provided to participants or used in intervention delivery or in training providers.	14-15	N/A
**4**	Procedures: Describe each of the procedures, activities, and/or processes used in the intervention including any enabling or support activities.	14-15	N/A
**5**	**WHO PROVIDED**<br>For each category of intervention provider (e.g. psychologist, nursing assistant), describe their expertise, background and any specific training given.	14-15	N/A
**6**	**HOW**<br>Describe the modes of delivery (e.g. face-to-face or by some other mechanism, such as internet or telephone) of the intervention and whether it was provided individually or in a group.	14-15	N/A
**7**	**WHERE**<br>Describe the type(s) of location(s) where the intervention occurred, including any necessary infrastructure or relevant features.	14-15	N/A
**8**	**WHEN AND HOW MUCH**<br>Describe the number of times the intervention was delivered and over what period of time including the number of sessions, their schedule, and their duration, intensity or dose.	14-15	N/A
**9**	**TAILORING**<br>If the intervention was planned to be personalised, titrated or adapted, then describe what, why when, and how.	14-15	N/A
**10**	**MODIFICATIONS**<br>If the intervention was modified during the course of the study, describe the changes (what, why, when, and how)	N/A	N/A
**11**	**HOW WELL**<br>Planned: If intervention adherence or fidelity was assessed, describe how and by whom, and if any strategies were used to maintain or improve fidelity, describe them.	9, 14-15	N/A
**12**	Actual: If intervention adherence or fidelity was assessed, describe the extent to which the intervention was delivered as planned.	N/A	N/A
