# Supplementary material for: Meniscal Transplant surgery or Optimised Rehabilitation full randomised trial (MeTeOR2): a study protocol
Source: BMJ Open. 2024 Jun 3;14(6):e085125. doi: 10.1136/bmjopen-2024-085125 (PMC11149128; doi:10.1136/bmjopen-2024-085125)
Supplement: Supplementary data [file bmjopen-2024-085125supp001.pdf]

Supplementary File 1: METEOR2 Consent Form_V4.0_01Aug2023

NIHR | National Institute for Health and Care Research — WARWICK — Meteor2 MENISCAL ALLOGRAFT TRIAL

METEOR2 Consent Form

Study: The meniscal transplant surgery or optimised rehabilitation

Chief Investigators: Professor Andrew Metcalfe & Professor Tim Spalding

**Participant ID:** ☐ ☐ ☐	**Please Initial:**
**1.** I confirm that I have read and understood the information sheet: (Version………………………… Date……………………………………) for the above study. I have had the opportunity to consider the information, ask questions and have had these answered satisfactorily.	
**2.** I understand that my participation is voluntary and that I am free to withdraw at any time, without giving any reason, without my medical care or legal rights being affected.	
**3.** I understand that relevant sections of any of my medical notes, information from my physiotherapists and data collected during the study may be looked at by responsible individuals from the Sponsors of the study, regulatory authorities or from the Health Trust, where it is relevant to my taking part in this research. I give permission for these individuals to have access to my records.	
**4.** I understand that appropriate personal identifying information will be collected via postal questionnaires or a mobile application and will be stored and used by the research team at Warwick Clinical Trials Unit or the relevant international coordinating centre to perform this study and enable the research team to contact me to follow up on my health status. This is on the understanding that any information will be treated with the strictest security and confidentiality.	
**5.** I understand that the information held and maintained by NHS England, other Central UK NHS bodies or other country specific equivalents may be used to help contact me or provide information about my health status.	
**6.** I agree to my GP being informed of my participation in the study and to be contacted for the collection of relevant health records to check for complications or issues related to my knee or operation.	☐ Yes<br>☐ No
**7.** I agree to being contacted either by text messages, email, telephone, post, or app notification by the METEOR2 Study Team to remind me when my questionnaire is due.	

NIHR | National Institute for Health and Care Research — WARWICK — Meteor2 MENISCAL ALLOGRAFT TRIAL

**8.** I agree to my contact details being kept for long-term follow up and to provide me with key study information such as study results.	
**9.** I understand that my anonymised data may be used for future ethically approved research.	
**10.** I agree to take part in the above study.	

Patient Name:	Signature:	Date:
Person Taking Consent:<br>Name:<br>Role:	Signature:	Date:

<u>For witnessed verbal consent:</u> I witnessed accurate reading of the consent form to the patient, who could ask any questions and was happy with the responses.

Witness Name & Role:	Witness Signature:	Date:

**16-17 Year Olds Only (Optional):**		
Guardians Name:	Guardians Signature:	Date:

FOR OFFICE USE ONLY

Copies ☐ 1 For Participant ☐ 1 for Hospital Notes ☐ Original document retained in site file.

Signature:
