# Supplementary material for: Meniscal Transplant surgery or Optimised Rehabilitation full randomised trial (MeTeOR2): a study protocol
Source: BMJ Open. 2024 Jun 3;14(6):e085125. doi: 10.1136/bmjopen-2024-085125 (PMC11149128; doi:10.1136/bmjopen-2024-085125)
Supplement: Author's manuscript [file bmjopen-2024-085125.draft_revisions.pdf]

BMJ Open

BMJ Open is committed to open peer review. As part of this commitment we make the peer review history of every article we publish publicly available.

When an article is published we post the peer reviewers' comments and the authors' responses online. We also post the versions of the paper that were used during peer review. These are the versions that the peer review comments apply to.

The versions of the paper that follow are the versions that were submitted during the peer review process. They are not the versions of record or the final published versions. They should not be cited or distributed as the published version of this manuscript.

BMJ Open is an open access journal and the full, final, typeset and author-corrected version of record of the manuscript is available on our site with no access controls, subscription charges or pay-per-view fees (http://bmjopen.bmj.com).

If you have any questions on BMJ Open's open peer review process please email info.bmjopen@bmj.com

BMJ Open

The Meniscal Transplant surgery or Optimised Rehabilitation full randomised trial (MeTeOR2): a study protocol

Journal:	*BMJ Open*
Manuscript ID	bmjopen-2024-085125
Article Type:	Protocol
Date Submitted by the Author:	06-Feb-2024
Complete List of Authors:	Arnold, Susanne; University of Warwick, Warwick Clinical Trials Unit<br>Spalding, Timothy; Cleveland Clinic London<br>Parsons, Helen; University of Warwick, Division of Health Sciences<br>Beard, David; University of Oxford, Nuffield Dept of Orthopaedics, Rheumatology and Musculoskeletal Sciences<br>Bradley, Helen; University of Warwick<br>Crisford, Peter; Patient Representative<br>Ellard, David; University of Warwick, Clinical Trials Unit;<br>Ferreira, Manuela; University of Sydney Institute of Bone and Joint Research,<br>Getgood, Alan; Western University,<br>Guck, Jonathan; University of Warwick<br>Haque, Aminul; University of Warwick, Warwick Clinical Trials Unit<br>Khan, Iftekhar; University of Warwick Warwick Medical School, Centre for Health Economics at Warwick<br>Mason, James; University of Warwick, Warwick Medical School<br>Milroy, Bryony; Patient Representative<br>Myers, P; Brisbane Orthopaedic & Sports Medicine Centre,<br>Parker, David; The University of Sydney Faculty of Medicine and Health<br>Price, Andrew; University of Oxford, Nuffield Department of Orthopaedics, Rheumatology and Musculoskeletal Sciences<br>Smith, Amy; Warwick Medical School<br>Smith, NA; University of Warwick, Clinical Sciences Research Laboratories<br>Smith, Toby; University of Warwick<br>Stewart, Kimberley; University of Warwick Warwick Medical School, Clinical Trials Unit<br>Underwood, Martin; Warwick University, Warwick Medical School<br>Verdonk, Peter; University of Antwerp<br>Metcalfe, Andrew; University of Warwick Warwick Medical School, Warwick Clinical Trials Unit; University Hospitals Coventry and Warwickshire NHS Trust, Trauma and Orthopaedics
Keywords:	Knee < ORTHOPAEDIC & TRAUMA SURGERY, Randomized Controlled Trial, REHABILITATION MEDICINE, SURGERY, TRANSPLANT SURGERY

SCHOLARONE™
Manuscripts

The Meniscal Transplant surgery or Optimised Rehabilitation full randomised trial (MeTeOR2): a study protocol

Susanne Arnold[1], Tim Spalding,[2] Helen Parsons,[1,3] David Beard,[4] Helen Bradley,[1] Peter Crisford,[5] David R Ellard[1,3], Manuela L Ferreira,[6] Alan Getgood,[7] Jonathan Guck,[1] Aminul Haque,[1] Iftekhar Khan,[1] James Mason,[1] Bryony Milroy,[5] Peter Myers,[8] David Parker,[6] Andrew Price,[9] Amy Smith,[1] Nick Smith,[3] Toby O Smith,[1,3] Kimberley Stewart,[1] Martin Underwood[1,3], Peter Verdonk,[10] Andrew Metcalfe[1,3]

[1]Warwick Clinical Trials Unit, University of Warwick, Coventry, UK.

[2]Cleveland Clinic, London, UK.

[3]University Hospital Coventry and Warwickshire NHS Trust, Coventry, UK.

[4]Oxford Surgical Intervention Trials Unit, University of Oxford, Oxford, UK.

[5]Patient representative

[6]The University of Sydney, Sydney Musculoskeletal Health, Faculty of Medicine and Health, Sydney, Australia.

[7]Western University, London, Canada.

[8]Brisbane Orthopaedic and Sports Medicine Centre, Brisbane, Australia.

[9]Nuffield Department of Orthopaedic Surgery, Oxford University.

[10]Univeristy of Antwerp, Antwerp, Belgium.

Corresponding author:

Andrew Metcalfe, Warwick Clinical Trials Unit, Warwick Medical School, University of Warwick, CV4 7AL, UK. Email: a.metcalfe@warwick.ac.uk

Word count (excluding title page, abstract, references and figures/tables): 4211

For peer review only

For peer review only - http://bmjopen.bmj.com/site/about/guidelines.xhtml

Abstract

Introduction
Pain and disability after meniscectomy can be a substantial lifelong problem. There are few treatment options, especially for young people. Non-surgical management (rehabilitation) is an option but increasingly surgeons are performing meniscal allograft transplants (MATs) for these individuals. However, this is still an uncommon procedure, and availability and usage of MAT varies widely both in the UK and internationally. It is not know which treatment option is the most effective and cost-effective.

Methods and analysis
The Meniscal Transplant surgery or Optimised Rehabilitation trial (MeTeOR2) is an international, multicentre, randomised controlled trial (RCT). The aim is to compare the clinical and cost-effectiveness of MAT versus an optimised package of individualised, progressive, rehabilitation that we have called Personalised Knee Therapy (PKT).

Participants will be recruited from sites across the UK, Australia, Canada and Belgium. The planned 144 participants provide at least 90% power to detect a 10-point difference in the Knee injury and Osteoarthritis Outcome Score (KOOS4) at 24-months post-randomisation (primary outcome). A prospectively planned economic evaluation will be conducted from a healthcare system and personal social services perspective. Secondary outcome data including health utility, occupational status, sports participation, mental wellbeing, further treatment, and adverse events will be collected at three, six, 12, 18, and 24 months. Analysis will be on an intention to treat basis and reported in-line with the Consolidated Standards of Reporting Trials statement.

Ethics and dissemination
The trial was approved by the London – Bloomsbury Research Ethics Committee on 19th August 2022 (22/LO/0327) and Northern Sydney Local Health District Human Research Ethics Committee, NSW, Australia on the 13th March 2023 (2022/ETH01890).

Trial results will be disseminated via peer-reviewed publications, presentations at international conferences, in lay summaries, and using social media as appropriate.

For peer review only

For peer review only - http://bmjopen.bmj.com/site/about/guidelines.xhtml

This protocol adheres to the recommended SPIRIT checklist.

Trial registration number: ISRCTN87336549

Strengths and limitations of this study

- International, multi-centre randomised controlled trial comparing an initial treatment strategy of MAT or Personalised Knee Therapy for adults with post-meniscectomy pain and functional loss.
- Participants will be recruited from sites across the UK, Australia, Canada and Belgium.
- Clinical outcomes assessed using the Knee Injury and Osteoarthritis Outcome Score (KOOS4) in addition to a range of secondary outcomes.
- Rare clinical presentation that may result in difficulty recruiting
- No radiological or biochemical measures to determine if MAT changes risk of osteoarthritis.

Keywords

Meniscectomy, Meniscal Allograft Transplant, Optimised Rehabilitation, Randomised Controlled Trial.

For peer review only

INTRODUCTION

A meniscectomy is an arthroscopic procedure where part or all of the meniscus is removed following an irreparable tear. Meniscal tears are common, often as a result of sporting injuries in young people.(1)

Every year, approximately 80,000 people in England undergo meniscectomy.(2) For the majority of people, pain and other symptoms such as locking, improve after surgery. However, some people have ongoing problems with pain and functional loss, leading to many years of disability. Ten years after a meniscectomy, 20% of people have developed osteoarthritis, increasing to 50% after 20 years.(3, 4) An individual aged 30-39 years having a partial meniscectomy is 40 times more likely to need a knee replacement after 15 years than that of the general population.(5)

There are few treatment options for people suffering with post-meniscectomy problems. Knee replacement is not usually recommended until people are over 50 years old. Therefore, younger adults with post-meniscectomy pain often have to live with their symptoms until they are at an age when knee replacement may be considered appropriate.(6, 7) Increasingly, orthopaedic surgeons are performing Meniscal Allograft Transplants (MATs) for people with post-meniscectomy pain.(8) Availability, access, and usage of MAT is, however, variable across the clinical community, resulting in a potential inequity in access to treatment. The transplant, which involves taking a donor meniscus from someone who has died and inserting it into someone with a similar size knee, is costly, at around £7,500 per case in the UK, although costs will vary substantially worldwide.

Non-surgical management (optimised rehabilitation) is also a viable treatment option.(9) The principles of care for early osteoarthritis could apply to this population, as rehabilitation has a strong evidence base in this (related) setting. Potential treatments include exercise, weight loss, lifestyle and activity advice, adjuncts such as orthoses and offloading braces, and non-steroidal anti-inflammatory drugs (NSAIDs); it is reasonable to think they could have a similar beneficial effect in the post-meniscectomy pain setting.(10-13) If shown to be effective for persistent pain after meniscectomy, a package of optimised rehabilitation would be safer, and cheaper, and does not impact on an individual’s ability to work or undertake other activities compared to a post-operative phase.

For peer review only - http://bmjopen.bmj.com/site/about/guidelines.xhtml

There is a lack of high-quality evidence about whether MAT or non-surgical care is more effective and which is the most cost-effective treatment for post-meniscectomy pain and functional loss. Our 2019 systematic review included 19 studies (N=1731); 18 case-series of MAT and just one randomised controlled trial (RCT) (this being a pilot study, N=21).(4) Patient reported outcomes improved from baseline across all studies. There are no data at present on non-surgical care for people with pain after meniscectomy, it would be very hard to make many meaningful comparison of a programme unless baseline differences between groups were balanced through randomisation.

Currently, there is no comparative data (except our previous pilot study)(14) to inform patients, clinicians, or health commissioners about whether MAT should be used or whether patients with post-meniscectomy pain would be better treated with non-surgical interventions.(4)

AIM

To compare the clinical and cost-effectiveness of an initial treatment strategy of MAT or Personalised Knee Therapy (an optimised package of rehabilitation) for adults with post-meniscectomy pain and functional loss.

For peer review only

METHODS AND ANALYSIS

Trial design

The Meniscal Transplant surgery or Optimised Rehabilitation (MeTeOR2) trial is an international, multicentre, two-arm RCT comparing the clinical and cost-effectiveness of MAT with an optimised package of rehabilitation termed PKT.

This paper and the trial protocol were written following the Standard Protocol Items: Recommendations for Interventional Trials (SPIRIT) guidelines. Figure 1 is the participant flow diagram, and a copy of the participant consent form is included as in supplementary file 1. A summary of core trial information is presented in the WHO trial registration data set in online supplementary file 2.

Patient and public involvement (PPI)

Patient involvement has been critical in the design and development of the trial and will continue to be important in its delivery, including the interpretation and dissemination of the results. As well as running several PPI events with patients who have experience of post-meniscectomy pain and either PKT or MAT, our research team includes two PPI representatives as co-applicants. Our PPI co-applicants are integral to the Trial Management Group (TMG), and are engaged in trial management meetings, contributing the patient perspective to trial processes and procedures and will be key to our dissemination plan. Two further people with post-meniscectomy pain sit on the Trial Steering Committee (TSC).

Objectives

Primary Objectives

- To compare the clinical effectiveness of an initial treatment strategy of MAT compared to PKT, based on participant-reported knee function at 24 months post-randomisation, using the four-domain version of the Knee Injury and Osteoarthritis Outcome Score (KOOS4).
- To determine the cost-effectiveness of MAT compared to PKT from an NHS and Personal, Social Service (PSS) perspective.

For peer review only - http://bmjopen.bmj.com/site/about/guidelines.xhtml

INSERT Figure 1 here

Figure 1. METEOR2 Participant Flow Diagram

Secondary objectives

- To quantify and draw inferences on, health utility, occupational status, sports participation, mental wellbeing, further treatment (including further surgery or physiotherapy in either arm) and adverse events (AEs) at three, six, 6, 12, 18, and 24 months.
- To evaluate process measures to compare days to initiation of treatment, rehabilitation attendance and participant expectation of outcome.

Outcome measures

Outcome measures were chosen in collaboration with our PPI representatives and our experienced clinical team. In line with SPIRIT guidance, Table 1 details the schedule of enrolment, interventions, and assessment.

Primary outcome

The primary clinical effectiveness outcome is the four domain **Knee Osteoarthritis Outcome Score (KOOS4)** score 24-months after randomisation.(15) This is a knee-specific instrument (0-100, 100 best score) incorporating four of the five domains of the full KOOS score (KOOS4 uses the domains for symptoms, pain, function/sports and quality of life, but not activities of daily living).(15) The KOOS4 has been widely used in previous trials of knee surgery including those with young adult, non-arthritic populations and is well-accepted by the clinical community.(14, 16-19)

Secondary Outcomes

All collected at six, 12, 18, and 24 months.

- The KOOS4 (also recorded at pre-intervention*).
- The five individual KOOS domains (also recorded at baseline). A validated knee specific instrument developed to assess the patients' opinion about their knee and associated problems.

For peer review only - http://bmjopen.bmj.com/site/about/guidelines.xhtml

- Health utility using EQ-5D-5L (also collected at baseline, pre-intervention* and three months).(20, 21)
- Short Warwick-Edinburgh Mental Wellbeing Scale (SWEMWBS, also collected at baseline).(22, 23)
- Tegner activity/sport scale (also collected at baseline).(24)
- Satisfaction with the outcome of treatment using a 5-point Likert scale.(25)
- The Participant Global Impression of Change. A single item, 7-point Likert scale.(26)
- The International Knee Documentation Committee (IKDC, also collected at baseline and 24 months).(27)
- Further knee surgery and physiotherapy.
- Resource use using participant questionnaires (also collected at three months).

*As an additional pre-intervention baseline (to understand any changes occurring whilst participants wait for their intervention, given the potential discrepancy between the groups), we will collect EQ-5D-5L and KOOS4 scores in the four weeks leading up to their intervention.

Safety outcomes

- Adverse events (AEs) and serious adverse events (SAEs) related to the surgery, anaesthetic, or rehabilitation will be collected according to relevant Warwick Clinical Trials Unit (WCTU) Standard Operating procedures (SOPs) at three, six, 12, 18, and 24-months post-randomisation.

Process and Fidelity Measures

- Days to initiation of randomised treatment, defined as the number of days between randomisation and the first physiotherapy contact for those who undergo PKT or the day of surgery.
- Physiotherapy attendance and intervention details will be recorded on physiotherapy case report forms (CRFs) at each appointment.
- A surgical CRF will be completed after each surgery including details about the anaesthetic and surgery such as surgical findings, surgical time, tourniquet time, graft size, fixation of graft and additional procedures.

For peer review only - http://bmjopen.bmj.com/site/about/guidelines.xhtml

Alongside a detailed data management plan, the programming team at Warwick Clinical Trials Unit (WCTU) have built a bespoke database management system to store and maintain high-quality data for the duration of the trial.

For peer review only

For peer review only - http://bmjopen.bmj.com/site/about/guidelines.xhtml

INSERT Table 1 here

Table 1. METEOR2 SPIRIT outcomes and assessment schedule

Eligibility criteria

Inclusion criteria:

1. Pain and/or functional restriction from the knee, severe enough to warrant potential MAT in the judgement of the treating clinician.

2. Previous meniscectomy ≥ six months ago.

Exclusion criteria:

1. Symptomatic ligament instability, not previously corrected, as determined by the assessing clinician.*

2. Coronal limb alignment which requires surgical correction, (previous correction, performed at least 6 months before entry to the trial, is not an exclusion criteria), as determined by the assessing clinician.*

3. Age under 16 years, or if ≥16 years, open growth plate at the proximal tibia as judged by the clinical team on imaging taken as part of standard care.

4. Full thickness cartilage loss (exposed bone) >1cm^2 on routine clinical magnetic resonance imaging (MRI), prior surgery, or any other form of clinical imaging or evaluation. This will be determined by the assessing clinician (it could be based on an assessment by a clinician or a radiologist, although the final decision rests with the treating clinician).

5. Inflammatory arthritis affecting the study knee as determined by the assessing clinician (i.e., a prior inflammatory event not considered to be related to the current clinical condition would not require exclusion).

6. Unable or unwilling to engage with rehabilitation.

7. Unable to adhere to trial processes.

8. Previous randomisation in the present trial (i.e., other knee). Where a previous randomisation has occurred in error, a participant may be withdrawn and this criterion will not apply.

For peer review only - http://bmjopen.bmj.com/site/about/guidelines.xhtml

*Previous surgery (except prior MAT) will be allowed. Where people have mal-alignment or ligament deficiency (30% of the MAT population), alignment or ligament deficiency may be corrected by osteotomy or ligament reconstruction, which should have been performed at least six months before entering someone into the study.

Participant identification, screening and withdrawals

Potential participants will be identified by clinical teams across the UK, Australia, Canada and Belgium. This will typically happen in outpatient or intermediate care clinics, hospital waiting lists, patient discharge lists, or referrals into hospital (from either primary, intermediate, or secondary care). Any potential participants will be assessed for eligibility by an appropriately delegated clinician. There is no requirement for any specific investigation although it is normal clinical practice to evaluate someone with a painful knee after prior meniscectomy with MRI scan for diagnostic purposes before any consideration of treatment options. This is likely to be the case for most participants but is not a requirement for entry. A screening log will be completed directly onto the trial database at all sites.

Those who are eligible will be given information about the trial and invited to discuss it further with a member of the local research team. They will be given adequate time to consider participation. Information sheets, invitation letters and other approved patient-facing materials may be posted, emailed, physically provided, or shared via other means to potential participants. A member of the local research team will carry out the informed consent process and baseline data collection.

Randomisation

After consent and baseline data have been collected, participants will be randomly allocated to the two treatment groups via a central computer-based randomisation system provided by WCTU, independent of the study team. Randomisation will be a 1:1 allocation determined by a minimisation algorithm using age (≥30 years or <30 years), treating site and knee compartment (lateral or medial) as factors, with a random factor included to provide a 70% weighting towards balance across the trial.

Participants will be randomised sequentially at site level. Randomisation will be performed by any member of the local clinical or research team on the delegation log, using the online system. Depending on local site arrangements, stickers,

For peer review only - http://bmjopen.bmj.com/site/about/guidelines.xhtml

electronic tags, or an equivalent may be used on the participant's clinical notes to flag their inclusion in the trial.

Randomised participants can choose to discontinue their treatment and/or withdraw from follow-up at any time, without prejudice. This will have no effect on their current or future care. All withdrawals will be monitored by the TMG and oversight committees.

Trial treatments(s)/interventions

Group 1: Meniscal Allograft Transplant (surgical intervention)

Participants allocated to surgery will receive MAT once an allograft becomes available. Surgical procedures are outlined in a trial-specific surgical manual which was developed at a surgical consensus meeting (July 2022) and informed by the International Meniscus Reconstruction Experts Forum (IMReF) guidelines.(8)

Immunosuppression is not required as meniscal allografts have low cellular content and are not rejected in the way solid organ transplants might be. Donors are screened for blood-borne diseases according to approved tissue bank policies. Essentially, this is similar to blood donors and there are no reported cases of viral transmission from MAT.

It is possible that participants may have to wait for six to 12 months for a graft of the right size to become available (median 6.5 months in our pilot study).(14) The delay to receive a graft is unavoidable in meniscal transplantation surgery, due to the availability of a suitable sized donor graft from tissue banks. To reduce time to surgery, the dimensions of the graft needed will be sent to tissue banks according to surgeon preference and usual practice. Recovery following MAT plateaus after nine to 12 months post-surgery. As the primary outcome is at 24 months, recovery will still be completed by the primary outcome time point, even for people in whom surgery is delayed.

All care, including the choice of anaesthetic, the surgical procedure, and post-operative analgesia, will be in accordance with usual procedures and care at participating sites.

For peer review only - http://bmjopen.bmj.com/site/about/guidelines.xhtml

Rehabilitation for the surgery group will be according to a standardised programme specific to MAT. We have used the lead centre's established programme for this and, in discussion with participating centres, adapted it to ensure it is deliverable across multiple NHS and international sites. A formal PKT programme will not be used prior to surgery in the MAT arm, although we do not discount people having prior or current physiotherapy.

Group 2: Personalised Knee Therapy (non-surgical intervention)

The PKT programme is a tailored and optimised package of rehabilitation aimed at improving individualised outcomes for people with knee pain and/or functional limitation following meniscectomy. It was developed from a pilot study,(14) and refined through literature and guideline reviews on non-surgical interventions including physiotherapy and other conservative interventions such as weight loss advice, knee braces and orthotics and referral to other services.(9-13, 28-30) This was supported with a consensus meeting held in July 2022, whereby experts in the field came together to develop a final comprehensive evidence-based PKT programme for the trial, ensuring it is deliverable across all of our UK and international study sites. A TIDieR checklist(31) for describing interventions for the METEOR2 PKT intervention is included as online supplemental file 3.

The PKT programme is outlined below:

Aim: To reduce pain, restore full knee range of motion, improve knee function and optimise overall social participation through a goal-setting approach personalised to the participant.

Delivered by: Physiotherapists trained in the principles of the METEOR2 PKT programme. Training includes the background to the problem of post-meniscectomy pain, the rationale for the trial, discussion about the PKT framework and trial administration procedures. Trained physiotherapists receive a comprehensive PKT manual containing a detailed account of all trial and intervention procedures.

Mode of delivery: As the intervention will be personalised to the participant, there is flexibility, as determined by clinical judgement and service provision, for PKT to be delivered face-to-face in acute hospital or community physiotherapy departments, through virtual consultation or a hybrid of the two.

For peer review only - http://bmjopen.bmj.com/site/about/guidelines.xhtml

Duration: Minimum of three months from first assessment and a minimum of four sessions in total, but can be as many as clinically required, reflecting normal clinical practice.

Treatment starting point from randomisation: When an appointment with an appropriate physiotherapist is available, according to normal clinical waiting times.

Timing of consultations: The interval between consultations will be personalised to the needs of the participant based on their presentation, progress and treatment goals.

Assessment: Initial assessment will include participant's history (subjective assessment) and physical examination (objective assessment). This will follow a routine musculoskeletal physiotherapy assessment. Specific focus in the objective assessment will be made on lower limb function and kinetic control, muscle length, strength and recruitment. Shared goals will be discussed between physiotherapist and participant which will form the basis of the problem list and treatment plan.

Treatments: Based on the individualised problem list and goals developed by the physiotherapist and participant, the physiotherapist will deliver interventions aimed to specifically manage the presenting problems. Through this, a personalised approach is made to the participants rehabilitation, optimising their outcome. Each participant will be provided with a manual which will include their treatment goals, information about their PKT programme with details of specific individualised exercises, and an exercise planner to encourage adherence.

End of trial

The trial will end when all 24-month follow-up data have been received and entered onto the trial database. The trial will only be stopped prior to this if mandated by the Research Ethics Committee (REC), following recommendation from the TSC, or if funding for the trial ceases.

The trial will be extended if we receive funding for five, 10, or 15–20-year follow-up. Consent will be obtained for long-term follow-up.

SAFETY REPORTING, AEs, SAEs

For peer review only - http://bmjopen.bmj.com/site/about/guidelines.xhtml

All Adverse Events (AEs) and Serious Adverse Events (SAEs) will be defined using widely accepted standard criteria. For this trial, AEs and SAEs will be collected from the point of randomisation onwards, up to 24-months. To avoid unnecessary reporting, some events which occur during treatment and recovery will be considered as normal aspects of the therapy, anaesthetic and post-operative recovery processes unless in the opinion of the clinical team, they are untoward, excessive or outside of what might normally be expected for the procedure; these will not need reporting. We will only collect AEs and SAEs related to the participants' knee, treatment they receive in the trial (or any treatment for the study knee) or trial processes.

SAEs will be reported to WCTU within 24-hours of the research staff becoming aware of the event. Events will be followed-up until they are resolved, or until the end of the trial, and an outcome has been agreed.

STATISTICAL ANALYSIS

Power and sample size

The target difference for KOOS4, widely used across multiple RCTs, is 10 points (on a 0-100 scale). This is consistent with anchor-based studies and is accepted as a clinically meaningful difference.(32-35) This effect is similar to that found for knee cartilage repair on autologous chondrocyte implantation.(32, 36) Cartilage repair was found to be clinically and cost-effective and it was subsequently recommended by the UK's National Institute for Health and Care Excellence (NICE).(37) MAT is about half the cost, so if it has a similar benefit it should be cost-effective.(36)

The pooled standard deviation (SD) in our pilot study was 14.5.(14) Allowing for the multicentre nature of this trial, along with the small size of the pilot, we have used the upper boundary of the 60% confidence interval to estimate the SD. According to the method of Chen et al,(38) this was 16.4. In this competency-based trial, each site will contribute small numbers and adjustment for clustering is not necessary. Hence, for a two-group parallel arm design, assuming 90% power and two-sided 5% significance, we would require a sample size of 116. Allowing for 20% loss to follow-up, the required sample size is 144.

Statistical analysis plan

For peer review only - http://bmjopen.bmj.com/site/about/guidelines.xhtml

All analyses will be reported in accordance with CONSORT guidelines.(39, 40) A comprehensive statistical analysis plan will be agreed with the Data Monitoring Committee (DMC) prior to any formal analysis. Descriptive statistics will be constructed for baseline data to check for any characteristic differences between allocation groups.

A generalised linear model will be used to assess differences in the KOOS4 at 24-months post-randomisation. As a minimum, adjustment terms for allocation, age and baseline score will be used. Variables found to be unbalanced at baseline may also be fitted, if judged appropriate. Where possible, a random effect for centre or country effects will also be used. Secondary outcomes will be analysed using a similar approach to the primary analysis where data type and distribution allows. Outcomes which are categorical in nature (e.g. patient global impression of change) will be analysed using proportional linear or logistic regression and subject to the same variable adjustments. To assess the effects of treatment switching, we will construct models to compare participants on an 'as treated' basis. That is, compare outcomes for those who received each treatment, regardless of allocated group. Exploratory models will be performed to assess the change from pre-intervention scores to the 24-month outcome. This may include assessing the trajectory of recovery over time using latent growth models, or assessing variables as prognostic or mediating factors. Pre-specified subgroup analyses will be performed for affected compartment (medial or lateral), age (30 years or over/under 30 years) and gender. Where possible, reasons for missing data will be ascertained and reported. If judged appropriate, effects of missing data will be explored using multiple imputation.

Database

All data will be stored in a customised database system developed by the experienced programming team at WCTU. This is supported by a detailed data management plan produced in accordance with WCTU SOPs to ensure high-quality data collection over the duration of the trial.

HEALTH ECONOMIC EVALUATION

A prospectively planned economic evaluation will be conducted from a health care system and personal social services perspective, according to the recommendations

For peer review only - http://bmjopen.bmj.com/site/about/guidelines.xhtml

of the NICE reference case.(41) Given multi-national recruitment, we will consider inter-country healthcare differences in constructing our analysis plan.(41, 42)

Health service contacts, made in connection with their treatments, will be recorded as part of the resource utilisation questionnaires at three, six, 12, 18, and 24-months. Time lost from work (paid/unpaid), will also be recorded. Participants will be encouraged to use an electronic or paper calendar to help recall this information at follow-up. Intervention and sequelae healthcare resource use will be costed using most recently available UK published national reference costs, reflated to a common year.(43, 44)

EQ-5D-5L scores will be converted to health status scores using the UK value set recommended by NICE guidance at the time of analysis.(45) Using the trapezoidal rule, the area-under-the-curve of health status scores will be calculated, providing patient-level QALY estimates. Quality of life years (QALYs) will be estimated for the whole cohort, applying UK values.

If the level of missingness data is below 5%, complete case analysis will be conducted. If not, mechanisms of missingness of data will be explored and multiple imputation methods will be applied to impute missing data. Complete case data or imputation sets will be used in bivariate analysis of costs and QALYs to generate incremental cost per QALY estimates and confidence intervals.(46-49) Findings will be analysed and visualised as cost-effectiveness acceptability curves, net monetary benefit and value of information analysis. The potential for heterogeneity of cost-effectiveness findings by country will be explored by fitting interaction terms to models, and if necessary performing country-specific analysis applying local costs to the complete clinical effectiveness data. A UK cohort-only analysis will be included within planned secondary analyses. If the pattern of costs and benefits is non-convergent or non-dominant at 24-months, we will develop a decision analytic model, using our expertise in economic modelling in this field.(4, 36, 50)

ETHICS AND DISSEMINATION

The trial was approved by the London – Bloomsbury Research Ethics Committee on 19th August 2022 (22/LO/0327) and Northern Sydney Local Health District Human Research Ethics Committee, NSW, Australia on 13th of March 2023 (2022/ETH01890). The trial will adhere to the Declaration of Helsinki and Good

For peer review only

Clinical Practice, following all relevant WCTU SOPs. Participants will provide informed consent before agreeing to participate. An independent DMC and TSC will provide oversight from set up to the closure of the trial. Using the definition provided by NIHR and WCTU SOPs, both committees will comprise independent members who will sign a separate committee charter. Data monitoring plans will be implemented by the trial sponsor. All protocol amendments will be communicated to sites by the trial co-ordinating team.

Data sharing

De-identified data that underlie the trial results will be available for non-commercial use, up to one year after publication of the primary outcome trial findings, or from metadata stored in a University repository up to ten years without investigator support. Third party access to trial data must be via a data-sharing agreement with the sponsors. They must have an ethically approved protocol in place for use of the data, and agree the approved protocol with the MeTeOR2 TMG. Data may be used for commercial purposes, according to the conditions above, but will need specific agreements in place prior to access being agreed, this may include a license fee. Analyses may include individual patient data meta-analyses or other purposes as agreed with the MeTeOR2 TMG.

Available data will include (but is not exclusive to) de-identified individual participant data, the study protocol, SAP, informed consent sheets and analytic codes used.

Trial registration and study timelines

The trial is registered with the ISRCTN register (ISRCTN87336549). The current version of the protocol is V2.0, approved on 24th November 2023. The planned dates of the study are from June 2022 to November 2027.

Dissemination and publication

Trial results will be shared with trial collaborators initially, with the main results paper being drafted by the trial team and agreed by the TSC before submission to a major peer-reviewed publication. Results will be disseminated at international meetings and conferences. Presenting results to patients and the public will be led in conjunction with our patient partners. Dissemination to trial participants will follow

For peer review only - http://bmjopen.bmj.com/site/about/guidelines.xhtml

current Health Research Authority (HRA) guidelines, with summaries provided on the METEOR2 website and social media as appropriate.

For peer review only

For peer review only - http://bmjopen.bmj.com/site/about/guidelines.xhtml

REFERENCES

1. Bhan K. Meniscal Tears: Current Understanding, Diagnosis, and Management. Cureus. 2020;12(6).
2. Abram SGF, Judge A, Beard DJ, Wilson HA, Price AJ. Temporal trends and regional variation in the rate of arthroscopic knee surgery in England: analysis of over 1.7 million procedures between 1997 and 2017. Has practice changed in response to new evidence? Br J Sports Med. 2019;53(24):1533-8.
3. Papalia R, Del Buono A, Osti L, Denaro V, Maffulli N. Meniscectomy as a risk factor for knee osteoarthritis: a systematic review. Br Med Bull. 2011;99:89-106.
4. Waugh N, Mistry H, Metcalfe A, Loveman E, Colquitt J, Royle P, et al. Meniscal allograft transplantation after meniscectomy: clinical effectiveness and cost-effectiveness. Knee Surg Sports Traumatol Arthrosc. 2019;27(6):1825-39.
5. Abram SGF, Judge A, Beard DJ, Carr AJ, Price AJ. Long-term rates of knee arthroplasty in a cohort of 834 393 patients with a history of arthroscopic partial meniscectomy. Bone Joint J. 2019;101-B(9):1071-80.
6. Bayliss LE, Culliford D, Monk AP, Glyn-Jones S, Prieto-Alhambra D, Judge A, et al. The effect of patient age at intervention on risk of implant revision after total replacement of the hip or knee: a population-based cohort study. Lancet. 2017;389(10077):1424-30.
7. Williams DP, Price AJ, Beard DJ, Hadfield SG, Arden NK, Murray DW, et al. The effects of age on patient-reported outcome measures in total knee replacements. Bone Joint J. 2013;95-B(1):38-44.
8. Getgood A, LaPrade RF, Verdonk P, Gersoff W, Cole B, Spalding T. International Meniscus Reconstruction Experts Forum (IMREF) 2015 Consensus Statement on the Practice of Meniscal Allograft Transplantation. Am J Sports Med. 2017;45(5):1195-205.
9. Ahmed I, Khatri C, Parsons N, Hutchinson CE, Staniszewska S, Price AJ, et al. Meniscal Tear Outcome (METRO) review: a protocol for a systematic review summarising the clinical course and patient experiences of meniscal tears in the current literature. BMJ Open. 2020;10(8):e036247.
10. Fransen M, McConnell S, Harmer AR, Van der Esch M, Simic M, Bennell KL. Exercise for osteoarthritis of the knee. Cochrane Database of Systematic Reviews. 2015;1.
11. NICE. Osteoarthritis in over 16s: diagnosis and management 2022 [Available from: https://www.nice.org.uk/guidance/ng226.
12. Bannuru RR, Osani MC, Vaysbrot EE, Arden NK, Bennell K, Bierma-Zeinstra SMA, et al. OARSI guidelines for the non-surgical management of knee, hip, and polyarticular osteoarthritis. Osteoarthritis Cartilage. 2019;27(11):1578-89.
13. Royal Australian College of General Practitioners. Guideline for the management of knee and hip osteoarthritis 2018 [Second:[Available from: https://www.racgp.org.au/getattachment/71ab5b77-afdf-4b01-90c3-04f61a910be6/Guideline-for-the-management-of-knee-and-hip-osteoarthritis.aspx.
14. Smith NA, Parsons N, Wright D, Hutchinson C, Metcalfe A, Thompson P, et al. A pilot randomized trial of meniscal allograft transplantation versus personalized physiotherapy for patients with a symptomatic meniscal deficient knee compartment. The Bone & Joint Journal. 2018;100:56-63.
15. Roos EM, Toksvig-Larsen S. Knee injury and Osteoarthritis Outcome Score (KOOS) - validation and comparison to the WOMAC in total knee replacement. Health Qual Life Outcomes. 2003;May 25(17).

For peer review only - http://bmjopen.bmj.com/site/about/guidelines.xhtml

16. Skou ST, Roos EM, Laursen MB, Rathleff MS, Arendt-Nielsen L, Simonsen O, et al. A Randomized, Controlled Trial of Total Knee Replacement. New England Journal of Medicine. 2015;373(17):1597-606.
17. Brittberg M, Recker D, Ilgenfritz J, Saris DBF, Group SES. Matrix-Applied Characterized Autologous Cultured Chondrocytes Versus Microfracture: Five-Year Follow-up of a Prospective Randomized Trial. American Journal of Sports Medicine. 2018;46(6):1343-51.
18. Frobell RB, Roos EM, Roos HP, Ranstam J, Lohmander LS. A randomized trial of treatment for acute anterior cruciate ligament tears. New England Journal of Medicine. 2010;363(4):331-42.
19. Davies L, Cook J, Leal J, Areia CM, Shirkey B, Jackson W, et al. Comparison of the clinical and cost effectiveness of two management strategies (rehabilitation versus surgical reconstruction) for non-acure anterior cruciate ligament (ACL) injury: study protocol for the ACL SNNAP randomised controlled trial. Trials. 2020;21(1).
20. EuroQol G. EuroQol--a new facility for the measurement of health-related quality of life. Health Policy. 1990;16(3):199-208.
21. Jin X, Al Sayah F, Ohinmaa A, Marshall DA, Johnson JA. Responsiveness of the EQ-5D-3L and EQ-5D-5L in patients following total hip or knee replacement. Qual Life Res. 2019;28(9):2409-17.
22. Stewart-Brown S, Tennant A, Tennant R, Platt S, Parkinson J, Weich S. Internal construct validity of the Warwick-Edinburgh Mental Well-being Scale (WEMWBS): a Rasch analysis using data from the Scottish Health Education Population Survey. Health Qual Life Outcomes. 2009;7(15).
23. Melendez-Torres GJ, Hewitt G, Hallingberg B, Anthony R, Collishaw S, Hall J, et al. Measurement invariance properties and external construct validity of the short Warwick-Edinburgh mental wellbeing scale in a large national sample of secondary school students in Wales. Health Qual Life Outcomes. 2019;17(139).
24. Tegner Y, Lysholm J. Rating systmes in the evaluation of knee ligament injuries. Clinical Orthopaedics & Related Research. 1985:43-9.
25. Rolfson O, Bohm E, Franklin P, Lyman S, Denissen G, Dawson J, et al. Patient-reported outcome measures in arthroplasty registries Report of the Patient-Reported Outcome Measures Working Group of the International Society of Arthroplasty Registries Part II. Recommendations for selection, administration, and analysis. Acta Orthop. 2016;87:9-23.
26. Kamper S, Maher C, Mackay G. Global rating of change scales: a review of strengths and weaknesses and considerations for design. J Man Manip Ther. 2009;17:163-70.
27. Irrgang J, Anderson A, Boland A, Harner C, Kurosaka M, Neyret P, et al. Development and validation of the international knee documentation committee subjective knee form. Am J Sports Med. 2001;29(5):600-13.
28. Kemp A, Hodgson B, Barnes L, Smith T. Predictors of the outcome of physiotherapy following a meniscus tear: A systematic review. Knee. 2021;33:125-42.
29. Filardo G, Kon E, Longo U, Madry H, Marchettini P, Marmotti A, et al. Non-surgical treatments for the management of early osteoarthritis. Knee Surg Sports Traumatol Arthrosc. 2016;24(6):1775-85.
30. Jones BQ, Covey CJ, Sineath MHJ. Nonsurgical Management of Knee Pain in Adults. Am Fam Physician. 2015;92(10):875-83.

For peer review only

For peer review only - http://bmjopen.bmj.com/site/about/guidelines.xhtml

31. Hoffmann TC, Glasziou PP, Boutron I, Milne R, Perera R, Moher D, et al. Better reporting of interventions: template for intervention description and replication (TIDieR) checklist and guide. BMJ. 2014;348:g1687.
32. Brittberg M, Recker D, Ilgenfritz J, Saris DBF, Group SES. Matrix-Applied Characterized Autologous Cultured Chondrocytes Versus Microfracture: Five-Year Follow-up of a Prospective Randomized Trial. Am J Sports Med. 2018;46(6):1343-51.
33. Frobell RB, Roos EM, Roos HP, Ranstam J, Lohmander LS. A randomized trial of treatment for acute anterior cruciate ligament tears. N Engl J Med. 2010;363(4):331-42.
34. Liu JN, Gowd AK, Redondo ML, Christian DR, Cabarcas BC, Yanke AB, et al. Establishing Clinically Significant Outcomes After Meniscal Allograft Transplantation. Orthop J Sports Med. 2019;7(1):2325967118818462.
35. Skou ST, Roos EM, Laursen MB, Rathleff MS, Arendt-Nielsen L, Simonsen O, et al. A Randomized, Controlled Trial of Total Knee Replacement. N Engl J Med. 2015;373(17):1597-606.
36. Mistry H, Connock M, Pink J, Shyangdan D, Clar C, Royle P, et al. Autologous chondrocyte implantation in the knee: systematic review and economic evaluation. Health Technology Assessment. 2017;21:1-294.
37. NICE. Autologous chondrocyte implantation for treating symptomatic articular cartilage defects of the knee 2017 [Available from: https://www.nice.org.uk/guidance/ta477.
38. Chen H, Zhang N, Lu X, Chen S. Caution regarding the choice of standard deviations to guide sample size calculations in clinical trials. Clin Trials. 2013;10(4):522-9.
39. Moher D, Hopewell S, Schulz KF, Montori V, Gotzsche PC, Devereaux PJ, et al. CONSORT 2010 statement: updated guidelines for reporting parallel group randomised trials. BMJ. 2010;340:c332.
40. Moher D, Schulz KF, Altman DG. The CONSORT statement: revised recommendations for improving the quality of reports of parallel-group randomised trials. Lancet. 2001;357(9263):1191-4.
41. National Institute for Health and Care Excellence. Guide to the methods of technology appraisal - Process and methods [PMG9]. 2013.
42. Dunn G, Emsley R, Liu H, Landau S, Green J, White I, et al. Evaluation and validation of social and psychological markers in randomised trials of complex interventions in mental health: a methodological research programme. Health Technol Assess. 2015;19(93):1-115, v-vi.
43. Curtis L, Burns A. Unit Costs of Health and Social Care 2015 Canterbury: Personal Social Services Research Unit, University of Kent; 2015 [Available from: https://www.pssru.ac.uk/project-pages/unit-costs/unit-costs-2015/.
44. Department of Health. Reference Costs 2015-16 Department of Health; 2016 [Available from: rnment/uploads/system/uploads/attachment_data/file/577083/Reference_Costs_2015-16.pdf.
45. NICE. Position statement on use of the EQ-5D-5L value set for England (updated October 2019). https://www.nice.org.uk/about/what-we-do/our-programmes/nice-guidance/technology-appraisal-guidance/eq-5d-5l. 2019.
46. Manca A, Hawkins N, Sculpher MJ. Estimating mean QALYs in trial-based cost-effectiveness analysis: the importance of controlling for baseline utility. Health Econ. 2005;14(5):487-96.

For peer review only

For peer review only - http://bmjopen.bmj.com/site/about/guidelines.xhtml

47. Sterne JA, White IR, Carlin JB, Spratt M, Royston P, Kenward MG, et al. Multiple imputation for missing data in epidemiological and clinical research: potential and pitfalls. BMJ. 2009;338:b2393.
48. White IR, Horton NJ, Carpenter J, Pocock SJ. Strategy for intention to treat analysis in randomised trials with missing outcome data. BMJ. 2011;342:d40.
49. White IR, Royston P, Wood AM. Multiple imputation using chained equations: Issues and guidance for practice. Stat Med. 2011;30(4):377-99.
50. Mistry H, Metcalfe A, Smith N, Loveman E, Colquitt J, Royle P, et al. The cost-effectiveness of osteochondral allograft transplantation in the knee. Knee Surg Sports Traumatol Arthrosc. 2019;27(6):1739-53.

For peer review only

For peer review only - http://bmjopen.bmj.com/site/about/guidelines.xhtml

Authors' contributions: AM and TS act as co-chief investigators and main grant holders for the METEOR2 study. HP, DB, DRE, MF AG, PM, DP, AP, NS, TOS, MU, & PV are co-applicant grant holders and are responsible for the design of METEOR2. These authors contributed to the development of the main trial protocol, the draft of this manuscript and approval of the final version. PC and BM are patient representatives as well as co-applicants on the grant and provided expertise from their perspectives. SA, TS, HP, DRE, MF, AG, AH, IK, JM, BM, PM, DP, AP, NS, TOS, MU, PV, HB, JG, AS, KS, and AM contributed to the development of the main trial protocol, in addition to contributing to the writing of the manuscript and approval of the final version. SA led the first draft of the manuscript, submitted the first draft of this manuscript and acted as corresponding author during the review process. This protocol was written following the SPIRIT protocol guidance.

Funding: METEOR2 is funded by the NIHR Health Technology Assessment programme (reference number: NIHR131629) and the Australian National Health and Medical Research Council (NHMRC) (Application ID: APP2007142; Type: National Institute for Health Research). The trial is sponsored by the University of Warwick.

Competing interests: AM, MU and HP are co-investigators on two current (RACER-Knee and RACER-Hip) and one completed (START:REACTS) NIHR funded studies (AM chief investigator of two) that have, or have had, additional support from Stryker Ltd. There is no link to the current trial.

AM, MU and HP are co-investigators on grants funded by the Australian NHMRC.

MU is chief investigator or co-investigator on multiple previous and current research grants from the UK National Institute for Health Research, and is a co-investigator on grants funded by the Norwegian MRC. He was an NIHR Senior Investigator until March 2021. He is a director and shareholder of Clinvivo Ltd that provides electronic data collection for health services research. He is part of an academic partnership with Serco Ltd, funded by the European Social Fund, related to return to work initiatives.

For peer review only

For peer review only - http://bmjopen.bmj.com/site/about/guidelines.xhtml

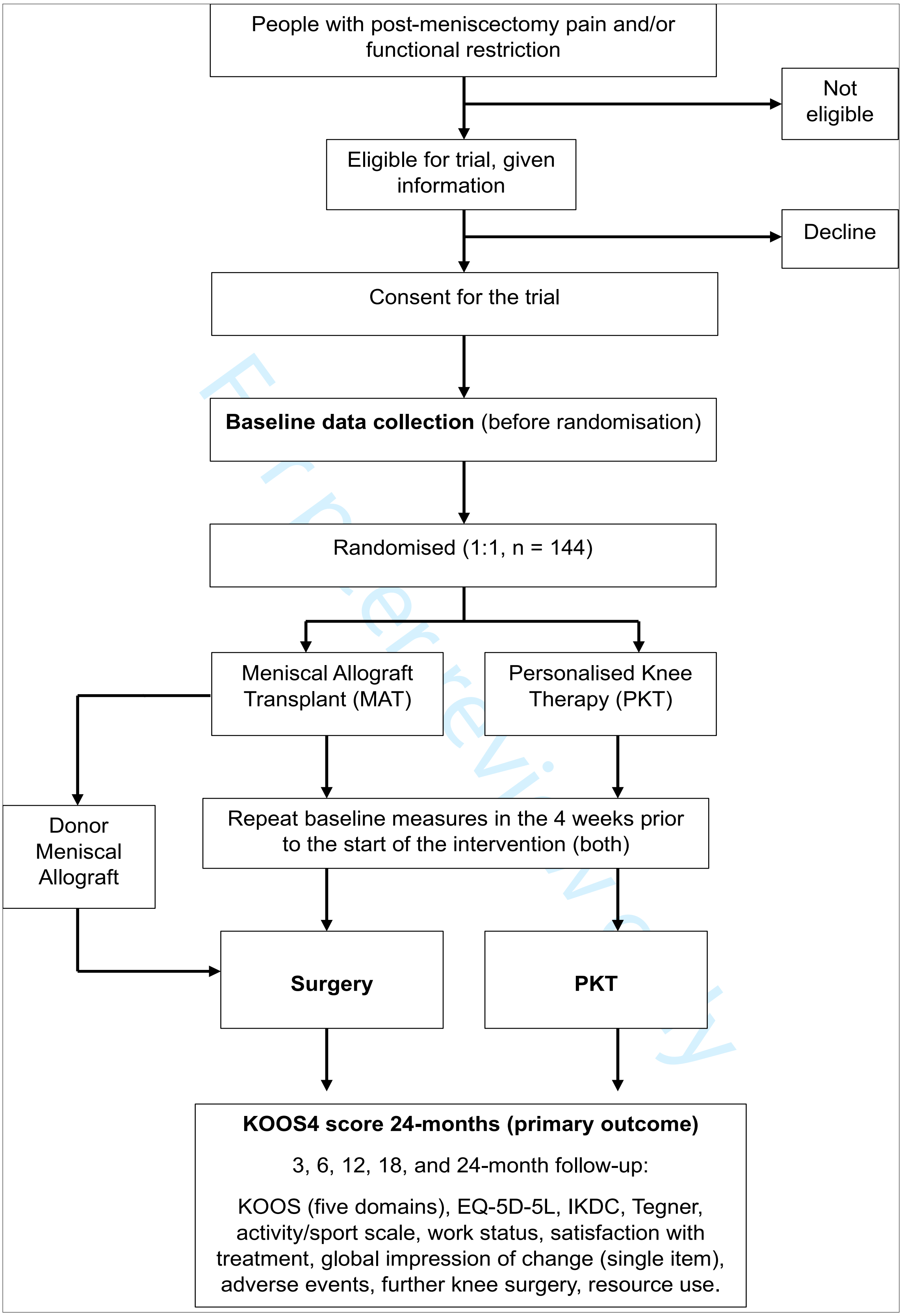

Figure 1. METEOR2 Participant Flow Diagram

KOOS, Knee injury and Osteoarthritis Outcome Score; EQ-5D-5L, EuroQol five dimensions; IKDC, International Knee Documentation Committee

Table 1. METEOR2 SPIRIT outcomes and assessment schedule

**Time point**	**-1**	**1**		**2**		**3**	**4**	**5**	**6**	**7**
Study period	Screening	Baseline	Pre-intervention	Intervention	Medical notes review	3-month	6-month	12-month	18-month	24-month
Check eligibility	✓									
Invitation to study	✓									
Informed consent		✓								
Medical history		✓								
Inclusion/exclusion criteria		✓								
Randomisation		✓								
Intervention (Surgery/PKT)				✓						
Operation note/Physio CRF				✓						
KOOS4		✓	✓				✓	✓	✓	✓
IKDC		✓	✓							✓
EQ-5D-5L		✓	✓			✓	✓	✓	✓	✓
Health resource use			✓			✓	✓	✓	✓	✓
SWEMWS		✓	✓				✓	✓	✓	✓
Tegner Activity Scale		✓	✓				✓	✓	✓	✓
Patient global impression of change							✓	✓	✓	✓
Complications, AEs, further surgery					✓	✓	✓	✓	✓	✓
Days to initiation of treatment				✓		✓	✓	✓	✓	
Rehabilitation attendance					✓	✓	✓	✓	✓	✓

For peer review only

METEOR2 SPIRIT 2013 checklist

Section/item	Item No	Description	Page Number
**Administrative information**			
Title	1	Descriptive title identifying the study design, population, interventions, and, if applicable, trial acronym	1
Trial registration	2a	Trial identifier and registry name. If not yet registered, name of intended registry	3
	2b	All items from the World Health Organization Trial Registration Data Set	Supp. File 2
Protocol version	3	Date and version identifier	19
Funding	4	Sources and types of financial, material, and other support	24
Roles and responsibilities	5a	Names, affiliations, and roles of protocol contributors	1, 20
	5b	Name and contact information for the trial sponsor	24
	5c	Role of study sponsor and funders, if any, in study design; collection, management, analysis, and interpretation of data; writing of the report; and the decision to submit the report for publication, including whether they will have ultimate authority over any of these activities	24
	5d	Composition, roles, and responsibilities of the coordinating centre, steering committee, endpoint adjudication committee, data management team, and other individuals or groups overseeing the trial, if applicable (see Item 21a for data monitoring committee)	24
**Introduction**			
Background and rationale	6a	Description of research question and justification for undertaking the trial, including summary of relevant studies (published and unpublished) examining benefits and harms for each intervention	4-5
	6b	Explanation for choice of comparators	4-5
Objectives	7	Specific objectives or hypotheses	6, 8

For peer review only

Trial design	8	Description of trial design including type of trial (e.g., parallel group, crossover, factorial, single group), allocation ratio, and framework (e.g., superiority, equivalence, noninferiority, exploratory)	6
**Methods: Participants, interventions, and outcomes**			
Study setting	9	Description of study settings (e.g., community clinic, academic hospital) and list of countries where data will be collected. Reference to where list of study sites can be obtained	12
Eligibility criteria	10	Inclusion and exclusion criteria for participants. If applicable, eligibility criteria for study centres and individuals who will perform the interventions (e.g., surgeons, psychotherapists)	11
Interventions	11a	Interventions for each group with sufficient detail to allow replication, including how and when they will be administered	13-15 & Supp. File 3
	11b	Criteria for discontinuing or modifying allocated interventions for a given trial participant (e.g., drug dose change in response to harms, participant request, or improving/worsening disease)	13-15 & Supp. File 3
	11c	Strategies to improve adherence to intervention protocols, and any procedures for monitoring adherence (e.g., drug tablet return, laboratory tests)	13-15 & Supp. File 3
	11d	Relevant concomitant care and interventions that are permitted or prohibited during the trial	13-15 & Supp. File 3
Outcomes	12	Primary, secondary, and other outcomes, including the specific measurement variable (e.g., systolic blood pressure), analysis metric (e.g., change from baseline, final value, time to event), method of aggregation (e.g., median, proportion), and time point for each outcome. Explanation of the clinical relevance of chosen efficacy and harm outcomes is strongly recommended	8-10
Participant timeline	13	Time schedule of enrolment, interventions (including any run-ins and washouts), assessments, and visits for participants. A schematic diagram is highly recommended (see Figure)	7

Sample size	14	Estimated number of participants needed to achieve study objectives and how it was determined, including clinical and statistical assumptions supporting any sample size calculations	16-17
Recruitment	15	Strategies for achieving adequate participant enrolment to reach target sample size	12

Methods: Assignment of interventions (for controlled trials)

Allocation:

Sequence generation	16a	Method of generating the allocation sequence (e.g., computer-generated random numbers), and list of any factors for stratification. To reduce predictability of a random sequence, details of any planned restriction (e.g., blocking) should be provided in a separate document that is unavailable to those who enrol participants or assign interventions	12
Allocation concealment mechanism	16b	Mechanism of implementing the allocation sequence (e.g., central telephone; sequentially numbered, opaque, sealed envelopes), describing any steps to conceal the sequence until interventions are assigned	12
Implementation	16c	Who will generate the allocation sequence, who will enrol participants, and who will assign participants to interventions	12
Blinding (masking)	17a	Who will be blinded after assignment to interventions (e.g., trial participants, care providers, outcome assessors, data analysts), and how	N/A
	17b	If blinded, circumstances under which unblinding is permissible, and procedure for revealing a participant's allocated intervention during the trial	N/A

Methods: Data collection, management, and analysis

Data collection methods	18a	Plans for assessment and collection of outcome, baseline, and other trial data, including any related processes to promote data quality (e.g., duplicate measurements, training of assessors) and a description of study instruments (e.g., questionnaires, laboratory tests) along with their reliability and validity, if known. Reference to where data collection forms can be found, if not in the protocol	8-9, 17

	18b	Plans to promote participant retention and complete follow-up, including list of any outcome data to be collected for participants who discontinue or deviate from intervention protocols	8-9,17
Data management	19	Plans for data entry, coding, security, and storage, including any related processes to promote data quality (e.g., double data entry; range checks for data values). Reference to where details of data management procedures can be found, if not in the protocol	17
Statistical methods	20a	Statistical methods for analysing primary and secondary outcomes. Reference to where other details of the statistical analysis plan can be found, if not in the protocol	16-18
	20b	Methods for any additional analyses (e.g., subgroup and adjusted analyses)	16-18
	20c	Definition of analysis population relating to protocol non-adherence (e.g., as randomised analysis), and any statistical methods to handle missing data (e.g., multiple imputation)	16-18
**Methods: Monitoring**			
Data monitoring	21a	Composition of data monitoring committee (DMC); summary of its role and reporting structure; statement of whether it is independent from the sponsor and competing interests; and reference to where further details about its charter can be found, if not in the protocol. Alternatively, an explanation of why a DMC is not needed	18
	21b	Description of any interim analyses and stopping guidelines, including who will have access to these interim results and make the final decision to terminate the trial	18
Harms	22	Plans for collecting, assessing, reporting, and managing solicited and spontaneously reported adverse events and other unintended effects of trial interventions or trial conduct	15-16
Auditing	23	Frequency and procedures for auditing trial conduct, if any, and whether the process will be independent from investigators and the sponsor	18
**Ethics and dissemination**			

Research ethics approval	24	Plans for seeking research ethics committee/institutional review board (REC/IRB) approval	2-3 & 18-19
Protocol amendments	25	Plans for communicating important protocol modifications (e.g., changes to eligibility criteria, outcomes, analyses) to relevant parties (e.g., investigators, REC/IRBs, trial participants, trial registries, journals, regulators)	18-19
Consent or assent	26a	Who will obtain informed consent or assent from potential trial participants or authorised surrogates, and how (see Item 32)	12
	26b	Additional consent provisions for collection and use of participant data and biological specimens in ancillary studies, if applicable	N/A
Confidentiality	27	How personal information about potential and enrolled participants will be collected, shared, and maintained in order to protect confidentiality before, during, and after the trial	17
Declaration of interests	28	Financial and other competing interests for principal investigators for the overall trial and each study site	24
Access to data	29	Statement of who will have access to the final trial dataset, and disclosure of contractual agreements that limit such access for investigators	19
Ancillary and post-trial care	30	Provisions, if any, for ancillary and post-trial care, and for compensation to those who suffer harm from trial participation	N/A
Dissemination policy	31a	Plans for investigators and sponsor to communicate trial results to participants, healthcare professionals, the public, and other relevant groups (e.g., via publication, reporting in results databases, or other data sharing arrangements), including any publication restrictions	2 & 19
	31b	Authorship eligibility guidelines and any intended use of professional writers	N/A
	31c	Plans, if any, for granting public access to the full protocol, participant-level dataset, and statistical code	19

Appendices

For peer review

Informed consent materials	32	Model consent form and other related documentation given to participants and authorised surrogates	Supp. File 1
Biological specimens	33	Plans for collection, laboratory evaluation, and storage of biological specimens for genetic or molecular analysis in the current trial and for future use in ancillary studies, if applicable	N/A

For peer review only

For peer review only

Supplementary File 1: METEOR2 Consent Form_V4.0_01Aug2023

NIHR | National Institute for Health and Care Research

WARWICK

Meteor2 MENISCAL ALLOGRAFT TRIAL

METEOR2 Consent Form

Study: The meniscal transplant surgery or optimised rehabilitation

Chief Investigators: Professor Andrew Metcalfe & Professor Tim Spalding

Participant ID: ☐ ☐ ☐	Please Initial:
**1.** I confirm that I have read and understood the information sheet: (Version……………………… Date…………………………………) for the above study. I have had the opportunity to consider the information, ask questions and have had these answered satisfactorily.	
**2.** I understand that my participation is voluntary and that I am free to withdraw at any time, without giving any reason, without my medical care or legal rights being affected.	
**3.** I understand that relevant sections of any of my medical notes, information from my physiotherapists and data collected during the study may be looked at by responsible individuals from the Sponsors of the study, regulatory authorities or from the Health Trust, where it is relevant to my taking part in this research. I give permission for these individuals to have access to my records.	
**4.** I understand that appropriate personal identifying information will be collected via postal questionnaires or a mobile application and will be stored and used by the research team at Warwick Clinical Trials Unit or the relevant international coordinating centre to perform this study and enable the research team to contact me to follow up on my health status. This is on the understanding that any information will be treated with the strictest security and confidentiality.	
**5.** I understand that the information held and maintained by NHS England, other Central UK NHS bodies or other country specific equivalents may be used to help contact me or provide information about my health status.	
**6.** I agree to my GP being informed of my participation in the study and to be contacted for the collection of relevant health records to check for complications or issues related to my knee or operation.	☐ Yes ☐ No
**7.** I agree to being contacted either by text messages, email, telephone, post, or app notification by the METEOR2 Study Team to remind me when my questionnaire is due.	

NIHR | National Institute for Health and Care Research

WARWICK

Meteor2 MENISCAL ALLOGRAFT TRIAL

**8.** I agree to my contact details being kept for long-term follow up and to provide me with key study information such as study results.	
**9.** I understand that my anonymised data may be used for future ethically approved research.	
**10.** I agree to take part in the above study.	

Patient Name:	Signature:	Date:
Person Taking Consent:<br>Name:<br>Role:	Signature:	Date:
<u>For witnessed verbal consent:</u> I witnessed accurate reading of the consent form to the patient, who could ask any questions and was happy with the responses.		
Witness Name & Role:	Witness Signature:	Date:

**16-17 Year Olds Only (Optional):**		
Guardians Name:	Guardians Signature:	Date:

FOR OFFICE USE ONLY

Copies ☐ 1 For Participant ☐ 1 for Hospital Notes ☐ Original document retained in site file.

Signature:

Supplementary File 2: METEOR2 WHO trial registration data set

Data Category	Information
Primary Registry and Trial Identifying Number	ISRCTN
Date of Registration in Primary Registry	02/09/2022
Secondary Identifying Numbers	NIHR HTA: 131629 , IRAS: 307686
Source(s) of Monetary or Material Support	National Institute for Health Research, Health Technology Assessment
Primary Sponsor	University of Warwick
Contact for Public Queries	meteor2@warwick.ac.uk
Contact for Scientific Queries	Mr Andrew Metcalfe (Co-CI), Warwick Clinical Trials Unit, University of Warwick.
Public Title	For people with pain after a meniscectomy, but without established OA, does a treatment strategy of undertaking MAT surgery or personalised knee therapy result in better clinical and/or cost effectiveness outcomes?
Scientific Title	The Meniscal Transplant Surgery or Optimised Rehabilitation - Full Randomised Controlled Trial
Countries of Recruitment	UK, Australia, Canada, Belgium.
Health Condition(s) or Problem(s) Studied	People with pain after meniscectomy but without established osteoarthritis
Intervention(s)	Surgical Intervention: Meniscal Allograft Transplant (MAT)<br>Non-surgical Intervention: Personalised Knee Therapy (PKT)
Key Inclusion and Exclusion Criteria	Inclusion criteria<br>i. Pain and/or functional restrictions from the knee, severe enough to warrant potential MAT in the judgement of the treating clinician.<br>ii. Previous meniscectomy more than 6 months ago.<br><br>Exclusion criteria:<br>i. Symptomatic ligament instability, not previously corrected, as determined by the assessing clinician.<br>ii. Coronal limb alignment which requires surgical correction, (previous correction, performed at least 6 months before entry to the trial, is not an exclusion criteria), as determined by the assessing clinician.<br>iii. Age under 16 years, or if ≥16, open growth plate at the proximal tibia as judged by the clinical team on imaging taken as part of standard care.<br>iv. Full thickness cartilage loss (exposed bone) >1 cm2 on routine clinical MRI, prior surgery, or any other form of clinical imaging or evaluation.

For peer review only

Data Category	Information
	v. Inflammatory arthritis affecting the study knee as determined by the assessing clinician.<br>vi. Unable or unwilling to engage with rehabilitation.<br>vii. Unable to adhere to trial processes.<br>viii. Previous randomisation in the present trial (i.e., other knee).
Study Type	Interventional<br>Allocation: randomised; individual assignment<br>Phase III
Date of First Enrolment	June 2023
Sample Size	144
Recruitment Status	Recruiting at time of submission
Primary Outcome(s)	1. Participant-reported knee function at 24 months, post randomisation, using the four-domain version of the Knee Injury and Osteoarthritis Outcome score (KOOS4).<br>2. To assess the cost effectiveness of MAT compared to PKT from an NHS and Personal, Social Service (PSS) perspective measured using health utility, occupational status, sports participant, mental wellbeing, further surgery (treatment switching or secondary knee surgery), satisfaction with the outcome of treatment, participant global impression of change and adverse events at three (EQ-5D 5L only), 6, 12, 18 and 24 months after randomisation
Key Secondary Outcomes	KOOS4, EQ-5D-5L, Short Warwick-Edinburgh Mental Wellbeing Scale, Tegner activity/sport scale, health resource use and analgesia use at baseline, pre-intervention, 6, 12, 18 and 24 months post randomisation.<br>International Knee Documentation Committee subjective score (IKDC) at baseline and 24 months post randomisation.<br>Satisfaction with the outcome of treatment, patient global impression of change, adverse events, further knee surgery at 6, 12, 18 and 24 months post randomisation.
Ethics Review	London – Bloomsbury Research Ethics Committee, August 2022.

For peer review only

Supplementary File 3: TIDieR Checklist for METEOR2 PKT Intervention

		Where located	
**Item number**	**Item**	**Primary paper (page number)**	**Other (details)**
**1**	**BRIEF NAME**<br>Provide the name or a phrase that describes the intervention	5, 14-15	N/A
**2**	**WHY**<br>Describe any rationale, theory, or goal of the elements essential to the intervention	14-15	N/A
**3**	**WHAT**<br>Materials: describe any physical or informational materials used in the intervention, including those provided to participants or used in intervention delivery or in training providers.	14-15	N/A
**4**	Procedures: Describe each of the procedures, activities, and/or processes used in the intervention including any enabling or support activities.	14-15	N/A
**5**	**WHO PROVIDED**<br>For each category of intervention provider (e.g. psychologist, nursing assistant), describe their expertise, background and any specific training given.	14-15	N/A
**6**	**HOW**<br>Describe the modes of delivery (e.g. face-to-face or by some other mechanism, such as internet or telephone) of the intervention and whether it was provided individually or in a group.	14-15	N/A
**7**	**WHERE**<br>Describe the type(s) of location(s) where the intervention occurred, including any necessary infrastructure or relevant features.	14-15	N/A
**8**	**WHEN AND HOW MUCH**<br>Describe the number of times the intervention was delivered and over what period of time including the number of sessions, their schedule, and their duration, intensity or dose.	14-15	N/A
**9**	**TAILORING**<br>If the intervention was planned to be personalised, titrated or adapted, then describe what, why when, and how.	14-15	N/A
**10**	**MODIFICATIONS**<br>If the intervention was modified during the course of the study, describe the changes (what, why, when, and how)	N/A	N/A
**11**	**HOW WELL**<br>Planned: If intervention adherence or fidelity was assessed, describe how and by whom, and if any strategies were used to maintain or improve fidelity, describe them.	9, 14-15	N/A
**12**	Actual: If intervention adherence or fidelity was assessed, describe the extent to which the intervention was delivered as planned.	N/A	N/A

For peer review only

BMJ Open

The Meniscal Transplant surgery or Optimised Rehabilitation full randomised trial (MeTeOR2): a study protocol

Journal:	*BMJ Open*
Manuscript ID	bmjopen-2024-085125.R1
Article Type:	Protocol
Date Submitted by the Author:	02-Apr-2024
Complete List of Authors:	Arnold, Susanne; University of Warwick, Warwick Clinical Trials Unit<br>Spalding, Timothy; Cleveland Clinic London<br>Parsons, Helen; University of Warwick, Division of Health Sciences<br>Beard, David; University of Oxford, Nuffield Dept of Orthopaedics, Rheumatology and Musculoskeletal Sciences<br>Bradley, Helen; University of Warwick<br>Crisford, Peter; Patient Representative<br>Ellard, David; University of Warwick, Clinical Trials Unit;<br>Ferreira, Manuela; University of Sydney Institute of Bone and Joint Research,<br>Getgood, Alan; Western University,<br>Guck, Jonathan; University of Warwick<br>Haque, Aminul; University of Warwick, Warwick Clinical Trials Unit<br>Khan, Iftekhar; University of Warwick Warwick Medical School, Centre for Health Economics at Warwick<br>Mason, James; University of Warwick, Warwick Medical School<br>Milroy, Bryony; Patient Representative<br>Myers, P; Brisbane Orthopaedic & Sports Medicine Centre,<br>Parker, David; The University of Sydney Faculty of Medicine and Health<br>Price, Andrew; University of Oxford, Nuffield Department of Orthopaedics, Rheumatology and Musculoskeletal Sciences<br>Smith, Amy; Warwick Medical School<br>Smith, NA; University of Warwick, Clinical Sciences Research Laboratories<br>Smith, Toby; University of Warwick<br>Stewart, Kimberley; University of Warwick Warwick Medical School, Clinical Trials Unit<br>Underwood, Martin; Warwick University, Warwick Medical School<br>Verdonk, Peter; University of Antwerp<br>Metcalfe, Andrew; University of Warwick Warwick Medical School, Warwick Clinical Trials Unit; University Hospitals Coventry and Warwickshire NHS Trust, Trauma and Orthopaedics
**Primary Subject Heading**:	Surgery
Secondary Subject Heading:	Sports and exercise medicine, Rehabilitation medicine
Keywords:	Knee < ORTHOPAEDIC & TRAUMA SURGERY, Randomized Controlled Trial, REHABILITATION MEDICINE, SURGERY, TRANSPLANT SURGERY

The Meniscal Transplant surgery or Optimised Rehabilitation full randomised trial (MeTeOR2): a study protocol

Susanne Arnold[1], Tim Spalding,[2] Helen Parsons,[1,3] David Beard,[4] Helen Bradley,[1] Peter Crisford,[5] David R Ellard[1,3], Manuela L Ferreira,[6] Alan Getgood,[7] Jonathan Guck,[1] Aminul Haque,[1] Iftekhar Khan,[1] James Mason,[1] Bryony Milroy,[5] Peter Myers,[8] David Parker,[6] Andrew Price,[9] Amy Smith,[1] Nick Smith,[3] Toby O Smith,[1,3] Kimberley Stewart,[1] Martin Underwood[1,3], Peter Verdonk,[10] Andrew Metcalfe[1,3]

[1]Warwick Clinical Trials Unit, University of Warwick, Coventry, UK.

[2]Cleveland Clinic, London, UK.

[3]University Hospital Coventry and Warwickshire NHS Trust, Coventry, UK.

[4]Oxford Surgical Intervention Trials Unit, University of Oxford, Oxford, UK.

[5]Patient representative

[6]The University of Sydney, Sydney Musculoskeletal Health, Faculty of Medicine and Health, Sydney, Australia.

[7]Western University, London, Canada.

[8]Brisbane Orthopaedic and Sports Medicine Centre, Brisbane, Australia.

[9]Nuffield Department of Orthopaedic Surgery, Oxford University.

[10]Univeristy of Antwerp, Antwerp, Belgium.

Corresponding author:

Andrew Metcalfe, Warwick Clinical Trials Unit, Warwick Medical School, University of Warwick, CV4 7AL, UK. Email: a.metcalfe@warwick.ac.uk

Word count (excluding title page, abstract, references and figures/tables): 4344

For peer review only - http://bmjopen.bmj.com/site/about/guidelines.xhtml

Abstract

Introduction

Pain and disability after meniscectomy can be a substantial lifelong problem. There are few treatment options, especially for young people. Non-surgical management (rehabilitation) is an option but increasingly surgeons are performing meniscal allograft transplants (MATs) for these individuals. However, this is still an uncommon procedure, and availability and usage of MAT varies widely both in the UK and internationally. It is not know which treatment option is the most effective and cost-effective.

Methods and analysis

The Meniscal Transplant surgery or Optimised Rehabilitation trial (MeTeOR2) is an international, multicentre, randomised controlled trial (RCT). The aim is to compare the clinical and cost-effectiveness of MAT versus an optimised package of individualised, progressive, rehabilitation that we have called Personalised Knee Therapy (PKT).

Participants will be recruited from sites across the UK, Australia, Canada and Belgium. The planned 144 participants provide at least 90% power to detect a 10-point difference in the Knee injury and Osteoarthritis Outcome Score (KOOS4) at 24-months post-randomisation (primary outcome). A prospectively planned economic evaluation will be conducted from a healthcare system and personal social services perspective. Secondary outcome data including health utility, occupational status, sports participation, mental wellbeing, further treatment, and adverse events will be collected at three, six, 12, 18, and 24 months. Analysis will be on an intention to treat basis and reported in-line with the Consolidated Standards of Reporting Trials statement.

Ethics and dissemination

The trial was approved by the London – Bloomsbury Research Ethics Committee on 19th August 2022 (22/LO/0327) and Northern Sydney Local Health District Human Research Ethics Committee, NSW, Australia on the 13th March 2023 (2022/ETH01890).

Trial results will be disseminated via peer-reviewed publications, presentations at international conferences, in lay summaries, and using social media as appropriate.

For peer review only

This protocol adheres to the recommended SPIRIT checklist.

Trial registration number: ISRCTN87336549

Strengths and limitations of this study

- International, multi-centre randomised controlled trial comparing an initial treatment strategy of MAT or Personalised Knee Therapy for adults with post-meniscectomy pain and functional loss.
- Participants will be recruited from sites across the UK, Australia, Canada and Belgium.
- Clinical outcomes assessed using the Knee Injury and Osteoarthritis Outcome Score (KOOS4) in addition to a range of secondary outcomes.
- Rare clinical presentation that may result in difficulty recruiting
- No radiological or biochemical measures to determine if MAT changes risk of osteoarthritis.

Keywords

Meniscectomy, Meniscal Allograft Transplant, Optimised Rehabilitation, Randomised Controlled Trial.

For peer review only

INTRODUCTION

A meniscectomy is an arthroscopic procedure where part or all of the meniscus is removed following an irreparable tear. Meniscal tears are common, often as a result of sporting injuries in young people.(1)

Every year, approximately 80,000 people in England undergo meniscectomy.(2) For the majority of people, pain and other symptoms such as locking, improve after surgery. However, some people have ongoing problems with pain and functional loss, leading to many years of disability. Ten years after a meniscectomy, 20% of people have developed osteoarthritis, increasing to 50% after 20 years.(3, 4) An individual aged 30-39 years having a partial meniscectomy is 40 times more likely to need a knee replacement after 15 years than that of the general population.(5)

There are few treatment options for people suffering with post-meniscectomy problems. Knee replacement is not usually recommended until people are over 50 years old. Therefore, younger adults with post-meniscectomy pain often have to live with their symptoms until they are at an age when knee replacement may be considered appropriate.(6, 7) Increasingly, orthopaedic surgeons are performing Meniscal Allograft Transplants (MATs) for people with post-meniscectomy pain.(8) Availability, access, and usage of MAT is, however, variable across the clinical community, resulting in a potential inequity in access to treatment. The transplant, which involves taking a donor meniscus from someone who has died and inserting it into someone with a similar size knee, is costly, at around £7,500 per case in the UK, although costs will vary substantially worldwide.

Non-surgical management (optimised rehabilitation) is also a viable treatment option.(9) The principles of care for early osteoarthritis could apply to this population, as rehabilitation has a strong evidence base in this (related) setting. Potential treatments include exercise, weight loss, lifestyle and activity advice, adjuncts such as orthoses and offloading braces, and non-steroidal anti-inflammatory drugs (NSAIDs); it is reasonable to think they could have a similar beneficial effect in the post-meniscectomy pain setting.(10-13) If shown to be effective for persistent pain after meniscectomy, a package of optimised rehabilitation would be safer, and cheaper, and does not impact on an individual’s ability to work or undertake other activities compared to a post-operative phase.

For peer review only - http://bmjopen.bmj.com/site/about/guidelines.xhtml

There is a lack of high-quality evidence about whether MAT or non-surgical care is more effective and which is the most cost-effective treatment for post-meniscectomy pain and functional loss. Our 2019 systematic review included 19 studies (N=1731); 18 case-series of MAT and just one randomised controlled trial (RCT) (this being a pilot study, N=21).(4) Patient reported outcomes improved from baseline across all studies. There are no data at present on non-surgical care for people with pain after meniscectomy, it would be very hard to make many meaningful comparison of a programme unless baseline differences between groups were balanced through randomisation.

Currently, there is no comparative data (except our previous pilot study)(14) to inform patients, clinicians, or health commissioners about whether MAT should be used or whether patients with post-meniscectomy pain would be better treated with non-surgical interventions.(4)

AIM

To compare the clinical and cost-effectiveness of an initial treatment strategy of MAT or Personalised Knee Therapy (an optimised package of rehabilitation) for adults with post-meniscectomy pain and functional loss.

For peer review only

METHODS AND ANALYSIS

Trial design

The Meniscal Transplant surgery or Optimised Rehabilitation (MeTeOR2) trial is an international, multicentre, two-arm RCT comparing the clinical and cost-effectiveness of MAT with an optimised package of rehabilitation termed PKT. The planned trial start date was June 2022. The planned trial end date is November 2027.

This paper and the trial protocol were written following the Standard Protocol Items: Recommendations for Interventional Trials (SPIRIT) guidelines. Figure 1 is the participant flow diagram, and a copy of the participant consent form is included as in supplementary file 1. A summary of core trial information is presented in the WHO trial registration data set in supplementary file 2.

Patient and public involvement (PPI)

Patient involvement has been critical in the design and development of the trial and will continue to be important in its delivery, including the interpretation and dissemination of the results. As well as running several PPI events with patients who have experience of post-meniscectomy pain and either PKT or MAT, our research team includes two PPI representatives as co-applicants. Our PPI co-applicants are integral to the Trial Management Group (TMG), and are engaged in trial management meetings, contributing the patient perspective to trial processes and procedures and will be key to our dissemination plan. Two further people with post-meniscectomy pain sit on the Trial Steering Committee (TSC).

Objectives

Primary Objectives

- To compare the clinical effectiveness of an initial treatment strategy of MAT compared to PKT, based on participant-reported knee function at 24 months post-randomisation, using the four-domain version of the Knee Injury and Osteoarthritis Outcome Score (KOOS4).
- To determine the cost-effectiveness of MAT compared to PKT from an NHS and Personal, Social Service (PSS) perspective.

For peer review only - http://bmjopen.bmj.com/site/about/guidelines.xhtml

INSERT Figure 1 here

Figure 1. METEOR2 Participant Flow Diagram

Secondary objectives

- To quantify and draw inferences on, health utility, occupational status, sports participation, mental wellbeing, further treatment (including further surgery or physiotherapy in either arm) and adverse events (AEs) at three, six, 6, 12, 18, and 24 months.
- To evaluate process measures to compare days to initiation of treatment, rehabilitation attendance and participant expectation of outcome.

Outcome measures

Outcome measures were chosen in collaboration with our PPI representatives and our experienced clinical team. In line with SPIRIT guidance, details of the schedule of enrolment, interventions, and assessment can be found in supplementary file 3.

Primary outcome

The primary clinical effectiveness outcome is participant-reported knee function using the four domain **Knee Osteoarthritis Outcome Score (KOOS4)** score 24-months after randomisation.(15) This is a knee-specific instrument (0-100, 100 best score) incorporating four of the five domains of the full KOOS score (KOOS4 uses the domains for symptoms, pain, function/sports and quality of life, but not activities of daily living).(15) The KOOS4 has been widely used in previous trials of knee surgery including those with young adult, non-arthritic populations and is well-accepted by the clinical community.(14, 16-19)

Cost effectiveness of the interventions will be assessed using health utility, occupational status, sports participation, mental wellbeing, further surgery (treatment switching or secondary knee surgery), satisfaction with the outcome of treatment, participant global impression of change and adverse events.

For peer review only - http://bmjopen.bmj.com/site/about/guidelines.xhtml

Secondary Outcomes

All collected at six, 12, 18, and 24 months.

- The KOOS4 (also recorded at pre-intervention*).
- The five individual KOOS domains (also recorded at baseline). A validated knee specific instrument developed to assess the patients' opinion about their knee and associated problems.
- Health utility using EQ-5D-5L (also collected at baseline, pre-intervention* and three months).(20, 21)
- Short Warwick-Edinburgh Mental Wellbeing Scale (SWEMWBS, also collected at baseline).(22, 23)
- Tegner activity/sport scale (also collected at baseline).(24)
- Satisfaction with the outcome of treatment using a 5-point Likert scale.(25)
- The Participant Global Impression of Change. A single item, 7-point Likert scale.(26)
- The International Knee Documentation Committee (IKDC, also collected at baseline and 24 months).(27)
- Further knee surgery and physiotherapy.
- Resource use using participant questionnaires (also collected at three months).
- Analgesia use.

*As an additional pre-intervention baseline (to understand any changes occurring whilst participants wait for their intervention, given the potential discrepancy between the groups), we will collect EQ-5D-5L and KOOS4 scores in the four weeks leading up to their intervention.

Safety outcomes

- Adverse events (AEs) and serious adverse events (SAEs) related to the surgery, anaesthetic, or rehabilitation will be collected according to relevant Warwick Clinical Trials Unit (WCTU) Standard Operating procedures (SOPs) at three, six, 12, 18, and 24-months post-randomisation.

Process and Fidelity Measures

For peer review only - http://bmjopen.bmj.com/site/about/guidelines.xhtml

- Days to initiation of randomised treatment, defined as the number of days between randomisation and the first physiotherapy contact for those who undergo PKT or the day of surgery.
- Physiotherapy attendance and intervention details will be recorded on physiotherapy case report forms (CRFs) at each appointment.
- A surgical CRF will be completed after each surgery including details about the anaesthetic and surgery such as surgical findings, surgical time, tourniquet time, graft size, fixation of graft and additional procedures.

Alongside a detailed data management plan, the programming team at Warwick Clinical Trials Unit (WCTU) have built a bespoke database management system to store and maintain high-quality data for the duration of the trial.

Eligibility criteria

Inclusion criteria:

1. Pain and/or functional restriction from the knee, severe enough to warrant potential MAT in the judgement of the treating clinician.

2. Previous meniscectomy ≥ six months ago.

Exclusion criteria:

1. Symptomatic ligament instability, not previously corrected, as determined by the assessing clinician.*

2. Coronal limb alignment which requires surgical correction, (previous correction, performed at least 6 months before entry to the trial, is not an exclusion criteria), as determined by the assessing clinician.*

3. Age under 16 years, or if ≥16 years, open growth plate at the proximal tibia as judged by the clinical team on imaging taken as part of standard care.

4. Full thickness cartilage loss (exposed bone) >1cm^2 on routine clinical magnetic resonance imaging (MRI), prior surgery, or any other form of clinical imaging or evaluation. This will be determined by the assessing clinician (it could be based on an assessment by a clinician or a radiologist, although the final decision rests with the treating clinician).

For peer review only - http://bmjopen.bmj.com/site/about/guidelines.xhtml

5. Inflammatory arthritis affecting the study knee as determined by the assessing clinician (i.e., a prior inflammatory event not considered to be related to the current clinical condition would not require exclusion).

6. Unable or unwilling to engage with rehabilitation.

7. Unable to adhere to trial processes.

8. Previous randomisation in the present trial (i.e., other knee). Where a previous randomisation has occurred in error, a participant may be withdrawn and this criterion will not apply.

*Previous surgery (except prior MAT) will be allowed. Where people have mal-alignment or ligament deficiency (30% of the MAT population), alignment or ligament deficiency may be corrected by osteotomy or ligament reconstruction, which should have been performed at least six months before entering someone into the study.

Participant identification, screening and withdrawals

Potential participants will be identified by clinical teams across the UK, Australia, Canada and Belgium. Currently, sites in the UK and Australia are recruiting participants (UK ethical approval obtained 22/08/2022 and open to recruitment 07/03/2023, Australian ethical approval obtained 04/04/2023 and open tor recruitment 08/12/2023). The centre in Canada has obtained ethical approval (20/11/2023) and plans to open to recruitment in Summer 2024. Ethical approval for sites in Belgium is pending but without a confirmed timeline yet.

Participant identification will typically happen in outpatient or intermediate care clinics, hospital waiting lists, patient discharge lists, or referrals into hospital (from either primary, intermediate, or secondary care). Any potential participants will be assessed for eligibility by an appropriately delegated clinician. There is no requirement for any specific investigation although it is normal clinical practice to evaluate someone with a painful knee after prior meniscectomy with MRI scan for diagnostic purposes before any consideration of treatment options. This is likely to be the case for most participants but is not a requirement for entry. A screening log will be completed directly onto the trial database at all sites.

Those who are eligible will be given information about the trial and invited to discuss it further with a member of the local research team. They will be given adequate time

For peer review only - http://bmjopen.bmj.com/site/about/guidelines.xhtml

For peer review only

to consider participation. Information sheets, invitation letters and other approved patient-facing materials may be posted, emailed, physically provided, or shared via other means to potential participants. A member of the local research team will carry out the informed consent process, collecting either written consent or witnessed remote verbal consent, and baseline data collection.

Randomisation

After consent and baseline data have been collected, participants will be randomly allocated to the two treatment groups via a central computer-based randomisation system provided by WCTU, independent of the study team. Randomisation will be a 1:1 allocation determined by a minimisation algorithm using age (≥30 years or <30 years), treating site and knee compartment (lateral or medial) as factors, with a random factor included to provide a 70% weighting towards balance across the trial.

Participants will be randomised sequentially at site level. Randomisation will be performed by any member of the local clinical or research team on the delegation log, using the online system. Depending on local site arrangements, stickers, electronic tags, or an equivalent may be used on the participant's clinical notes to flag their inclusion in the trial.

Randomised participants can choose to discontinue their treatment and/or withdraw from follow-up at any time, without prejudice. This will have no effect on their current or future care. All withdrawals will be monitored by the TMG and oversight committees.

Trial treatments(s)/interventions

Group 1: Meniscal Allograft Transplant (surgical intervention)

Participants allocated to surgery will receive MAT once an allograft becomes available. Surgical procedures are outlined in a trial-specific surgical manual which was developed at a surgical consensus meeting (July 2022) and informed by the International Meniscus Reconstruction Experts Forum (IMReF) guidelines.(8)

Immunosuppression is not required as meniscal allografts have low cellular content and are not rejected in the way solid organ transplants might be. Donors are screened for blood-borne diseases according to approved tissue bank policies.

For peer review only - http://bmjopen.bmj.com/site/about/guidelines.xhtml

Essentially, this is similar to blood donors and there are no reported cases of viral transmission from MAT.

It is possible that participants may have to wait for six to 12 months for a graft of the right size to become available (median 6.5 months in our pilot study).(14) The delay to receive a graft is unavoidable in meniscal transplantation surgery, due to the availability of a suitable sized donor graft from tissue banks. To reduce time to surgery, the dimensions of the graft needed will be sent to tissue banks according to surgeon preference and usual practice. Recovery following MAT plateaus after nine to 12 months post-surgery. As the primary outcome is at 24 months, recovery will still be completed by the primary outcome time point, even for people in whom surgery is delayed.

All care, including the choice of anaesthetic, the surgical procedure, and post-operative analgesia, will be in accordance with usual procedures and care at participating sites.

Rehabilitation for the surgery group will be according to a standardised programme specific to MAT. We have used the lead centre's established programme for this and, in discussion with participating centres, adapted it to ensure it is deliverable across multiple NHS and international sites. A formal PKT programme will not be used prior to surgery in the MAT arm, although we do not discount people having prior or current physiotherapy.

Group 2: Personalised Knee Therapy (non-surgical intervention)

The PKT programme is a tailored and optimised package of rehabilitation aimed at improving individualised outcomes for people with knee pain and/or functional limitation following meniscectomy. It was developed from a pilot study,(14) and refined through literature and guideline reviews on non-surgical interventions including physiotherapy and other conservative interventions such as weight loss advice, knee braces and orthotics and referral to other services.(9-13, 28-30) This was supported with a consensus meeting held in July 2022, whereby experts in the field came together to develop a final comprehensive evidence-based PKT programme for the trial, ensuring it is deliverable across all of our UK and

For peer review only - http://bmjopen.bmj.com/site/about/guidelines.xhtml

international study sites. A TIDieR checklist(31) for describing interventions for the METEOR2 PKT intervention is included as supplementary file 4.

The PKT programme is outlined below:

Aim: To reduce pain, restore full knee range of motion, improve knee function and optimise overall social participation through a goal-setting approach personalised to the participant.

Delivered by: Physiotherapists trained in the principles of the METEOR2 PKT programme. Training includes the background to the problem of post-meniscectomy pain, the rationale for the trial, discussion about the PKT framework and trial administration procedures. Trained physiotherapists receive a comprehensive PKT manual containing a detailed account of all trial and intervention procedures.

Mode of delivery: As the intervention will be personalised to the participant, there is flexibility, as determined by clinical judgement and service provision, for PKT to be delivered face-to-face in acute hospital or community physiotherapy departments, through virtual consultation or a hybrid of the two.

Duration: Minimum of three months from first assessment and a minimum of four sessions in total, but can be as many as clinically required, reflecting normal clinical practice.

Treatment starting point from randomisation: When an appointment with an appropriate physiotherapist is available, according to normal clinical waiting times.

Timing of consultations: The interval between consultations will be personalised to the needs of the participant based on their presentation, progress and treatment goals.

Assessment: Initial assessment will include participant's history (subjective assessment) and physical examination (objective assessment). This will follow a routine musculoskeletal physiotherapy assessment. Specific focus in the objective assessment will be made on lower limb function and kinetic control, muscle length, strength and recruitment. Shared goals will be discussed between physiotherapist and participant which will form the basis of the problem list and treatment plan.

For peer review only - http://bmjopen.bmj.com/site/about/guidelines.xhtml

Treatments: Based on the individualised problem list and goals developed by the physiotherapist and participant, the physiotherapist will deliver interventions aimed to specifically manage the presenting problems. Through this, a personalised approach is made to the participants rehabilitation, optimising their outcome. Each participant will be provided with a manual which will include their treatment goals, information about their PKT programme with details of specific individualised exercises, and an exercise planner to encourage adherence.

End of trial

The trial will end when all 24-month follow-up data have been received and entered onto the trial database. The trial will only be stopped prior to this if mandated by the Research Ethics Committee (REC), following recommendation from the TSC, or if funding for the trial ceases.

The trial will be extended if we receive funding for five, 10, or 15–20-year follow-up. Consent will be obtained for long-term follow-up.

SAFETY REPORTING, AEs, SAEs

All Adverse Events (AEs) and Serious Adverse Events (SAEs) will be defined using widely accepted standard criteria. For this trial, AEs and SAEs will be collected from the point of randomisation onwards, up to 24-months. To avoid unnecessary reporting, some events which occur during treatment and recovery will be considered as normal aspects of the therapy, anaesthetic and post-operative recovery processes unless in the opinion of the clinical team, they are untoward, excessive or outside of what might normally be expected for the procedure; these will not need reporting. We will only collect AEs and SAEs related to the participants' knee, treatment they receive in the trial (or any treatment for the study knee) or trial processes.

SAEs will be reported to WCTU within 24-hours of the research staff becoming aware of the event. Events will be followed-up until they are resolved, or until the end of the trial, and an outcome has been agreed.

STATISTICAL ANALYSIS

Power and sample size

For peer review only - http://bmjopen.bmj.com/site/about/guidelines.xhtml

The target difference for KOOS4, widely used across multiple RCTs, is 10 points (on a 0-100 scale). This is consistent with anchor-based studies and is accepted as a clinically meaningful difference.(32-35) This effect is similar to that found for knee cartilage repair on autologous chondrocyte implantation.(32, 36) Cartilage repair was found to be clinically and cost-effective and it was subsequently recommended by the UK's National Institute for Health and Care Excellence (NICE).(37) MAT is about half the cost, so if it has a similar benefit it should be cost-effective.(36)

The pooled standard deviation (SD) in our pilot study was 14.5.(14) Allowing for the multicentre nature of this trial, along with the small size of the pilot, we have used the upper boundary of the 60% confidence interval to estimate the SD. According to the method of Chen et al,(38) this was 16.4. In this competency-based trial, each site will contribute small numbers and adjustment for clustering is not necessary. Hence, for a two-group parallel arm design, assuming 90% power and two-sided 5% significance, we would require a sample size of 116. Allowing for 20% loss to follow-up, the required sample size is 144.

Statistical analysis plan

All analyses will be reported in accordance with CONSORT guidelines.(39, 40) A comprehensive statistical analysis plan will be agreed with the Data Monitoring Committee (DMC) prior to any formal analysis. Descriptive statistics will be constructed for baseline data to check for any characteristic differences between allocation groups.

A generalised linear model will be used to assess differences in the KOOS4 at 24-months post-randomisation. As a minimum, adjustment terms for allocation, age and baseline score will be used. Variables found to be unbalanced at baseline may also be fitted, if judged appropriate. Where possible, a random effect for centre or country effects will also be used. Secondary outcomes will be analysed using a similar approach to the primary analysis where data type and distribution allows. Outcomes which are categorical in nature (e.g. patient global impression of change) will be analysed using proportional linear or logistic regression and subject to the same variable adjustments. To assess the effects of treatment switching, we will construct models to compare participants on an 'as treated' basis. That is, compare outcomes for those who received each treatment, regardless of allocated group. Exploratory

For peer review only - http://bmjopen.bmj.com/site/about/guidelines.xhtml

models will be performed to assess the change from pre-intervention scores to the 24-month outcome. This may include assessing the trajectory of recovery over time using latent growth models, or assessing variables as prognostic or mediating factors. Pre-specified subgroup analyses will be performed for affected compartment (medial or lateral), age (30 years or over/under 30 years) and gender. Where possible, reasons for missing data will be ascertained and reported. If judged appropriate, effects of missing data will be explored using multiple imputation.

Database

All data will be stored in a customised database system developed by the experienced programming team at WCTU. This is supported by a detailed data management plan produced in accordance with WCTU SOPs to ensure high-quality data collection over the duration of the trial.

HEALTH ECONOMIC EVALUATION

A prospectively planned economic evaluation will be conducted from a health care system and personal social services perspective, according to the recommendations of the NICE reference case.(41) Given multi-national recruitment, we will consider inter-country healthcare differences in constructing our analysis plan.(41, 42)

Health service contacts, made in connection with their treatments, will be recorded as part of the resource utilisation questionnaires at three, six, 12, 18, and 24-months. Time lost from work (paid/unpaid), will also be recorded. Participants will be encouraged to use an electronic or paper calendar to help recall this information at follow-up. Intervention and sequelae healthcare resource use will be costed using most recently available UK published national reference costs, reflated to a common year.(43, 44)

EQ-5D-5L scores will be converted to health status scores using the UK value set recommended by NICE guidance at the time of analysis.(45) Using the trapezoidal rule, the area-under-the-curve of health status scores will be calculated, providing patient-level QALY estimates. Quality of life years (QALYs) will be estimated for the whole cohort, applying UK values.

If the level of missingness data is below 5%, complete case analysis will be conducted. If not, mechanisms of missingness of data will be explored and multiple

For peer review only - http://bmjopen.bmj.com/site/about/guidelines.xhtml

imputation methods will be applied to impute missing data. Complete case data or imputation sets will be used in bivariate analysis of costs and QALYs to generate incremental cost per QALY estimates and confidence intervals.(46-49) Findings will be analysed and visualised as cost-effectiveness acceptability curves, net monetary benefit and value of information analysis. The potential for heterogeneity of cost-effectiveness findings by country will be explored by fitting interaction terms to models, and if necessary performing country-specific analysis applying local costs to the complete clinical effectiveness data. A UK cohort-only analysis will be included within planned secondary analyses. If the pattern of costs and benefits is non-convergent or non-dominant at 24-months, we will develop a decision analytic model, using our expertise in economic modelling in this field.(4, 36, 50)

ETHICS AND DISSEMINATION

The trial was approved by the London – Bloomsbury Research Ethics Committee on 19th August 2022 (22/LO/0327) and Northern Sydney Local Health District Human Research Ethics Committee, NSW, Australia on 13th of March 2023 (2022/ETH01890). The trial will adhere to the Declaration of Helsinki and Good Clinical Practice, following all relevant WCTU SOPs. Participants will provide informed consent before agreeing to participate. An independent DMC and TSC will provide oversight from set up to the closure of the trial. Using the definition provided by NIHR and WCTU SOPs, both committees will comprise independent members who will sign a separate committee charter. Data monitoring plans will be implemented by the trial sponsor. All protocol amendments will be communicated to sites by the trial co-ordinating team.

Data sharing

De-identified data that underlie the trial results will be available for non-commercial use, up to one year after publication of the primary outcome trial findings, or from metadata stored in a University repository up to ten years without investigator support. Third party access to trial data must be via a data-sharing agreement with the sponsors. They must have an ethically approved protocol in place for use of the data, and agree the approved protocol with the MeTeOR2 TMG. Data may be used for commercial purposes, according to the conditions above, but will need specific agreements in place prior to access being agreed, this may include a license fee.

For peer review only - http://bmjopen.bmj.com/site/about/guidelines.xhtml

Analyses may include individual patient data meta-analyses or other purposes as agreed with the MeTeOR2 TMG.

Available data will include (but is not exclusive to) de-identified individual participant data, the study protocol, SAP, informed consent sheets and analytic codes used.

Trial registration and study timelines

The trial is registered with the ISRCTN register (ISRCTN87336549). The current version of the protocol is V2.0, approved on 24th November 2023. The planned dates of the study are from June 2022 to November 2027.

Dissemination and publication

Trial results will be shared with trial collaborators initially, with the main results paper being drafted by the trial team and agreed by the TSC before submission to a major peer-reviewed publication. Results will be disseminated at international meetings and conferences. Presenting results to patients and the public will be led in conjunction with our patient partners. Dissemination to trial participants will follow current Health Research Authority (HRA) guidelines, with summaries provided on the METEOR2 website and social media as appropriate.

For peer review only

For peer review only - http://bmjopen.bmj.com/site/about/guidelines.xhtml

REFERENCES

1. Bhan K. Meniscal Tears: Current Understanding, Diagnosis, and Management. Cureus. 2020;12(6).
2. Abram SGF, Judge A, Beard DJ, Wilson HA, Price AJ. Temporal trends and regional variation in the rate of arthroscopic knee surgery in England: analysis of over 1.7 million procedures between 1997 and 2017. Has practice changed in response to new evidence? Br J Sports Med. 2019;53(24):1533-8.
3. Papalia R, Del Buono A, Osti L, Denaro V, Maffulli N. Meniscectomy as a risk factor for knee osteoarthritis: a systematic review. Br Med Bull. 2011;99:89-106.
4. Waugh N, Mistry H, Metcalfe A, Loveman E, Colquitt J, Royle P, et al. Meniscal allograft transplantation after meniscectomy: clinical effectiveness and cost-effectiveness. Knee Surg Sports Traumatol Arthrosc. 2019;27(6):1825-39.
5. Abram SGF, Judge A, Beard DJ, Carr AJ, Price AJ. Long-term rates of knee arthroplasty in a cohort of 834 393 patients with a history of arthroscopic partial meniscectomy. Bone Joint J. 2019;101-B(9):1071-80.
6. Bayliss LE, Culliford D, Monk AP, Glyn-Jones S, Prieto-Alhambra D, Judge A, et al. The effect of patient age at intervention on risk of implant revision after total replacement of the hip or knee: a population-based cohort study. Lancet. 2017;389(10077):1424-30.
7. Williams DP, Price AJ, Beard DJ, Hadfield SG, Arden NK, Murray DW, et al. The effects of age on patient-reported outcome measures in total knee replacements. Bone Joint J. 2013;95-B(1):38-44.
8. Getgood A, LaPrade RF, Verdonk P, Gersoff W, Cole B, Spalding T. International Meniscus Reconstruction Experts Forum (IMREF) 2015 Consensus Statement on the Practice of Meniscal Allograft Transplantation. Am J Sports Med. 2017;45(5):1195-205.
9. Ahmed I, Khatri C, Parsons N, Hutchinson CE, Staniszewska S, Price AJ, et al. Meniscal Tear Outcome (METRO) review: a protocol for a systematic review summarising the clinical course and patient experiences of meniscal tears in the current literature. BMJ Open. 2020;10(8):e036247.
10. Fransen M, McConnell S, Harmer AR, Van der Esch M, Simic M, Bennell KL. Exercise for osteoarthritis of the knee. Cochrane Database of Systematic Reviews. 2015;1.
11. NICE. Osteoarthritis in over 16s: diagnosis and management 2022 [Available from: https://www.nice.org.uk/guidance/ng226.
12. Bannuru RR, Osani MC, Vaysbrot EE, Arden NK, Bennell K, Bierma-Zeinstra SMA, et al. OARSI guidelines for the non-surgical management of knee, hip, and polyarticular osteoarthritis. Osteoarthritis Cartilage. 2019;27(11):1578-89.
13. Royal Australian College of General Practitioners. Guideline for the management of knee and hip osteoarthritis 2018 [Second:[Available from: https://www.racgp.org.au/getattachment/71ab5b77-afdf-4b01-90c3-04f61a910be6/Guideline-for-the-management-of-knee-and-hip-osteoarthritis.aspx.
14. Smith NA, Parsons N, Wright D, Hutchinson C, Metcalfe A, Thompson P, et al. A pilot randomized trial of meniscal allograft transplantation versus personalized physiotherapy for patients with a symptomatic meniscal deficient knee compartment. The Bone & Joint Journal. 2018;100:56-63.
15. Roos EM, Toksvig-Larsen S. Knee injury and Osteoarthritis Outcome Score (KOOS) - validation and comparison to the WOMAC in total knee replacement. Health Qual Life Outcomes. 2003;May 25(17).

For peer review only - http://bmjopen.bmj.com/site/about/guidelines.xhtml

16. Skou ST, Roos EM, Laursen MB, Rathleff MS, Arendt-Nielsen L, Simonsen O, et al. A Randomized, Controlled Trial of Total Knee Replacement. New England Journal of Medicine. 2015;373(17):1597-606.
17. Brittberg M, Recker D, Ilgenfritz J, Saris DBF, Group SES. Matrix-Applied Characterized Autologous Cultured Chondrocytes Versus Microfracture: Five-Year Follow-up of a Prospective Randomized Trial. American Journal of Sports Medicine. 2018;46(6):1343-51.
18. Frobell RB, Roos EM, Roos HP, Ranstam J, Lohmander LS. A randomized trial of treatment for acute anterior cruciate ligament tears. New England Journal of Medicine. 2010;363(4):331-42.
19. Davies L, Cook J, Leal J, Areia CM, Shirkey B, Jackson W, et al. Comparison of the clinical and cost effectiveness of two management strategies (rehabilitation versus surgical reconstruction) for non-acure anterior cruciate ligament (ACL) injury: study protocol for the ACL SNNAP randomised controlled trial. Trials. 2020;21(1).
20. EuroQol G. EuroQol--a new facility for the measurement of health-related quality of life. Health Policy. 1990;16(3):199-208.
21. Jin X, Al Sayah F, Ohinmaa A, Marshall DA, Johnson JA. Responsiveness of the EQ-5D-3L and EQ-5D-5L in patients following total hip or knee replacement. Qual Life Res. 2019;28(9):2409-17.
22. Stewart-Brown S, Tennant A, Tennant R, Platt S, Parkinson J, Weich S. Internal construct validity of the Warwick-Edinburgh Mental Well-being Scale (WEMWBS): a Rasch analysis using data from the Scottish Health Education Population Survey. Health Qual Life Outcomes. 2009;7(15).
23. Melendez-Torres GJ, Hewitt G, Hallingberg B, Anthony R, Collishaw S, Hall J, et al. Measurement invariance properties and external construct validity of the short Warwick-Edinburgh mental wellbeing scale in a large national sample of secondary school students in Wales. Health Qual Life Outcomes. 2019;17(139).
24. Tegner Y, Lysholm J. Rating systmes in the evaluation of knee ligament injuries. Clinical Orthopaedics & Related Research. 1985:43-9.
25. Rolfson O, Bohm E, Franklin P, Lyman S, Denissen G, Dawson J, et al. Patient-reported outcome measures in arthroplasty registries Report of the Patient-Reported Outcome Measures Working Group of the International Society of Arthroplasty Registries Part II. Recommendations for selection, administration, and analysis. Acta Orthop. 2016;87:9-23.
26. Kamper S, Maher C, Mackay G. Global rating of change scales: a review of strengths and weaknesses and considerations for design. J Man Manip Ther. 2009;17:163-70.
27. Irrgang J, Anderson A, Boland A, Harner C, Kurosaka M, Neyret P, et al. Development and validation of the international knee documentation committee subjective knee form. Am J Sports Med. 2001;29(5):600-13.
28. Kemp A, Hodgson B, Barnes L, Smith T. Predictors of the outcome of physiotherapy following a meniscus tear: A systematic review. Knee. 2021;33:125-42.
29. Filardo G, Kon E, Longo U, Madry H, Marchettini P, Marmotti A, et al. Non-surgical treatments for the management of early osteoarthritis. Knee Surg Sports Traumatol Arthrosc. 2016;24(6):1775-85.
30. Jones BQ, Covey CJ, Sineath MHJ. Nonsurgical Management of Knee Pain in Adults. Am Fam Physician. 2015;92(10):875-83.

For peer review only - http://bmjopen.bmj.com/site/about/guidelines.xhtml

31. Hoffmann TC, Glasziou PP, Boutron I, Milne R, Perera R, Moher D, et al. Better reporting of interventions: template for intervention description and replication (TIDieR) checklist and guide. BMJ. 2014;348:g1687.
32. Brittberg M, Recker D, Ilgenfritz J, Saris DBF, Group SES. Matrix-Applied Characterized Autologous Cultured Chondrocytes Versus Microfracture: Five-Year Follow-up of a Prospective Randomized Trial. Am J Sports Med. 2018;46(6):1343-51.
33. Frobell RB, Roos EM, Roos HP, Ranstam J, Lohmander LS. A randomized trial of treatment for acute anterior cruciate ligament tears. N Engl J Med. 2010;363(4):331-42.
34. Liu JN, Gowd AK, Redondo ML, Christian DR, Cabarcas BC, Yanke AB, et al. Establishing Clinically Significant Outcomes After Meniscal Allograft Transplantation. Orthop J Sports Med. 2019;7(1):2325967118818462.
35. Skou ST, Roos EM, Laursen MB, Rathleff MS, Arendt-Nielsen L, Simonsen O, et al. A Randomized, Controlled Trial of Total Knee Replacement. N Engl J Med. 2015;373(17):1597-606.
36. Mistry H, Connock M, Pink J, Shyangdan D, Clar C, Royle P, et al. Autologous chondrocyte implantation in the knee: systematic review and economic evaluation. Health Technology Assessment. 2017;21:1-294.
37. NICE. Autologous chondrocyte implantation for treating symptomatic articular cartilage defects of the knee 2017 [Available from: https://www.nice.org.uk/guidance/ta477.
38. Chen H, Zhang N, Lu X, Chen S. Caution regarding the choice of standard deviations to guide sample size calculations in clinical trials. Clin Trials. 2013;10(4):522-9.
39. Moher D, Hopewell S, Schulz KF, Montori V, Gotzsche PC, Devereaux PJ, et al. CONSORT 2010 statement: updated guidelines for reporting parallel group randomised trials. BMJ. 2010;340:c332.
40. Moher D, Schulz KF, Altman DG. The CONSORT statement: revised recommendations for improving the quality of reports of parallel-group randomised trials. Lancet. 2001;357(9263):1191-4.
41. National Institute for Health and Care Excellence. Guide to the methods of technology appraisal - Process and methods [PMG9]. 2013.
42. Dunn G, Emsley R, Liu H, Landau S, Green J, White I, et al. Evaluation and validation of social and psychological markers in randomised trials of complex interventions in mental health: a methodological research programme. Health Technol Assess. 2015;19(93):1-115, v-vi.
43. Curtis L, Burns A. Unit Costs of Health and Social Care 2015 Canterbury: Personal Social Services Research Unit, University of Kent; 2015 [Available from: https://www.pssru.ac.uk/project-pages/unit-costs/unit-costs-2015/.
44. Department of Health. Reference Costs 2015-16 Department of Health; 2016 [Available from: rnment/uploads/system/uploads/attachment_data/file/577083/Reference_Costs_2015-16.pdf.
45. NICE. Position statement on use of the EQ-5D-5L value set for England (updated October 2019). https://www.nice.org.uk/about/what-we-do/our-programmes/nice-guidance/technology-appraisal-guidance/eq-5d-5l. 2019.
46. Manca A, Hawkins N, Sculpher MJ. Estimating mean QALYs in trial-based cost-effectiveness analysis: the importance of controlling for baseline utility. Health Econ. 2005;14(5):487-96.

For peer review only - http://bmjopen.bmj.com/site/about/guidelines.xhtml

47. Sterne JA, White IR, Carlin JB, Spratt M, Royston P, Kenward MG, et al. Multiple imputation for missing data in epidemiological and clinical research: potential and pitfalls. BMJ. 2009;338:b2393.
48. White IR, Horton NJ, Carpenter J, Pocock SJ. Strategy for intention to treat analysis in randomised trials with missing outcome data. BMJ. 2011;342:d40.
49. White IR, Royston P, Wood AM. Multiple imputation using chained equations: Issues and guidance for practice. Stat Med. 2011;30(4):377-99.
50. Mistry H, Metcalfe A, Smith N, Loveman E, Colquitt J, Royle P, et al. The cost-effectiveness of osteochondral allograft transplantation in the knee. Knee Surg Sports Traumatol Arthrosc. 2019;27(6):1739-53.

For peer review only

For peer review only - http://bmjopen.bmj.com/site/about/guidelines.xhtml

Authors' contributions: AM and TS act as co-chief investigators and main grant holders for the METEOR2 study. Alongside the following co-applicant grant holders, HP, DB, DRE, MLF, AG, JM, PM, DP, AP, NS, TOS, MU, & PV. they are responsible for the design of METEOR2 and the main trial protocol. They provided expertise in orthopaedic surgery, physiotherapy, statistics, health economics, and clinical trial methodology. All of these authors contributed to the planning and drafting of this manuscript, and gave approval of the final version.

PC and BM are patient representatives as well as co-applicants on the grant. They contributed to this manuscript, providing expertise and input from the patient perspectives and gave approval of the final version.

SA, JG, AH, IK, AS, and KS contributed to the development of the main trial protocol. They provided expertise in physiotherapy, statistics, health economics, and trial management to contribute to planning and writing this manuscript and gave approval of the final version.

SA led the first draft of the manuscript, submitted the first draft of this manuscript and acted as corresponding author during the review process.

AM will be corresponding author for post-publication communication.

This protocol was written following the SPIRIT protocol guidance.

Funding: METEOR2 is funded by the NIHR Health Technology Assessment programme (reference number: NIHR131629) and the Australian National Health and Medical Research Council (NHMRC) (Application ID: APP2007142; Type: National Institute for Health Research). The trial is sponsored by the University of Warwick.

Competing interests: AM, MU and HP are co-investigators on two current (RACER-Knee and RACER-Hip) and one completed (START:REACTS) NIHR funded studies (AM chief investigator of two) that have, or have had, additional support from Stryker Ltd. There is no link to the current trial.

AM, MU and HP are co-investigators on grants funded by the Australian NHMRC.

MU is chief investigator or co-investigator on multiple previous and current research grants from the UK National Institute for Health Research, and is a co-investigator on

For peer review only - http://bmjopen.bmj.com/site/about/guidelines.xhtml

grants funded by the Norwegian MRC. He was an NIHR Senior Investigator until March 2021. He is a director and shareholder of Clinvivo Ltd that provides electronic data collection for health services research. He is part of an academic partnership with Serco Ltd, funded by the European Social Fund, related to return to work initiatives.

For peer review only

For peer review only - http://bmjopen.bmj.com/site/about/guidelines.xhtml

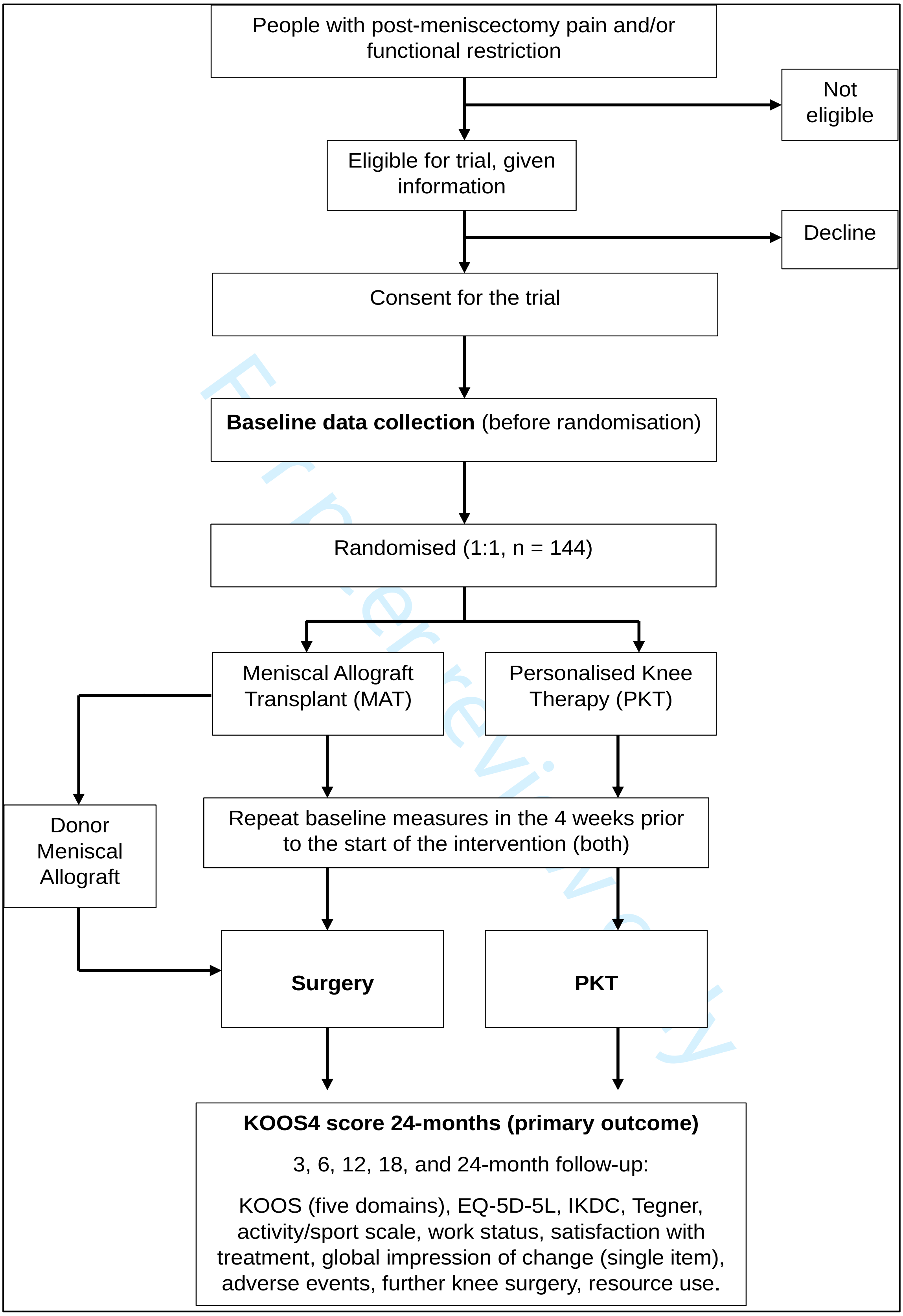

Figure 1. METEOR2 Participant Flow Diagram

KOOS, Knee injury and Osteoarthritis Outcome Score; EQ-5D-5L, EuroQol five dimensions; IKDC, International Knee Documentation Committee

For peer review only - http://bmjopen.bmj.com/site/about/guidelines.xhtml

Supplementary File 1: METEOR2 Consent Form_V4.0_01Aug2023

NIHR | National Institute for Health and Care Research   WARWICK   Meteor2 MENISCAL ALLOGRAFT TRIAL

METEOR2 Consent Form

Study: The meniscal transplant surgery or optimised rehabilitation

Chief Investigators: Professor Andrew Metcalfe & Professor Tim Spalding

**Participant ID:** ☐ ☐ ☐	**Please Initial:**
**1.** I confirm that I have read and understood the information sheet: (Version.............................. Date...) for the above study. I have had the opportunity to consider the information, ask questions and have had these answered satisfactorily.	
**2.** I understand that my participation is voluntary and that I am free to withdraw at any time, without giving any reason, without my medical care or legal rights being affected.	
**3.** I understand that relevant sections of any of my medical notes, information from my physiotherapists and data collected during the study may be looked at by responsible individuals from the Sponsors of the study, regulatory authorities or from the Health Trust, where it is relevant to my taking part in this research. I give permission for these individuals to have access to my records.	
**4.** I understand that appropriate personal identifying information will be collected via postal questionnaires or a mobile application and will be stored and used by the research team at Warwick Clinical Trials Unit or the relevant international coordinating centre to perform this study and enable the research team to contact me to follow up on my health status. This is on the understanding that any information will be treated with the strictest security and confidentiality.	
**5.** I understand that the information held and maintained by NHS England, other Central UK NHS bodies or other country specific equivalents may be used to help contact me or provide information about my health status.	
**6.** I agree to my GP being informed of my participation in the study and to be contacted for the collection of relevant health records to check for complications or issues related to my knee or operation.	☐ Yes<br>☐ No
**7.** I agree to being contacted either by text messages, email, telephone, post, or app notification by the METEOR2 Study Team to remind me when my questionnaire is due.	

NIHR | National Institute for Health and Care Research   WARWICK   Meteor2 MENISCAL ALLOGRAFT TRIAL

**8.** I agree to my contact details being kept for long-term follow up and to provide me with key study information such as study results.	
**9.** I understand that my anonymised data may be used for future ethically approved research.	
**10.** I agree to take part in the above study.	

Patient Name:	Signature:	Date:
Person Taking Consent:<br>Name:<br>Role:	Signature:	Date:
For witnessed verbal consent: I witnessed accurate reading of the consent form to the patient, who could ask any questions and was happy with the responses.		
Witness Name & Role:	Witness Signature:	Date:

**16-17 Year Olds Only (Optional):**		
Guardians Name:	Guardians Signature:	Date:

FOR OFFICE USE ONLY

Copies ☐ 1 For Participant ☐ 1 for Hospital Notes ☐ Original document retained in site file.

Signature:

Supplementary File 2: METEOR2 WHO trial registration data set

Data Category	Information
Primary Registry and Trial Identifying Number	ISRCTN
Date of Registration in Primary Registry	02/09/2022
Secondary Identifying Numbers	NIHR HTA: 131629 , IRAS: 307686
Source(s) of Monetary or Material Support	National Institute for Health Research, Health Technology Assessment
Primary Sponsor	University of Warwick
Contact for Public Queries	meteor2@warwick.ac.uk
Contact for Scientific Queries	Mr Andrew Metcalfe (Co-CI), Warwick Clinical Trials Unit, University of Warwick.
Public Title	For people with pain after a meniscectomy, but without established OA, does a treatment strategy of undertaking MAT surgery or personalised knee therapy result in better clinical and/or cost effectiveness outcomes?
Scientific Title	The Meniscal Transplant Surgery or Optimised Rehabilitation - Full Randomised Controlled Trial
Countries of Recruitment	UK, Australia, Canada, Belgium.
Health Condition(s) or Problem(s) Studied	People with pain after meniscectomy but without established osteoarthritis
Intervention(s)	Surgical Intervention: Meniscal Allograft Transplant (MAT)<br>Non-surgical Intervention: Personalised Knee Therapy (PKT)
Key Inclusion and Exclusion Criteria	Inclusion criteria<br>i. Pain and/or functional restrictions from the knee, severe enough to warrant potential MAT in the judgement of the treating clinician.<br>ii. Previous meniscectomy more than 6 months ago.<br><br>Exclusion criteria:<br>i. Symptomatic ligament instability, not previously corrected, as determined by the assessing clinician.<br>ii. Coronal limb alignment which requires surgical correction, (previous correction, performed at least 6 months before entry to the trial, is not an exclusion criteria), as determined by the assessing clinician.<br>iii. Age under 16 years, or if ≥16, open growth plate at the proximal tibia as judged by the clinical team on imaging taken as part of standard care.<br>iv. Full thickness cartilage loss (exposed bone) >1 cm2 on routine clinical MRI, prior surgery, or any other form of clinical imaging or evaluation.

For peer review only

Data Category	Information
	v. Inflammatory arthritis affecting the study knee as determined by the assessing clinician.<br>vi. Unable or unwilling to engage with rehabilitation.<br>vii. Unable to adhere to trial processes.<br>viii. Previous randomisation in the present trial (i.e., other knee).
Study Type	Interventional<br>Allocation: randomised; individual assignment<br>Phase III
Date of First Enrolment	June 2023
Sample Size	144
Recruitment Status	Recruiting at time of submission
Primary Outcome(s)	1. Participant-reported knee function at 24 months, post randomisation, using the four-domain version of the Knee Injury and Osteoarthritis Outcome score (KOOS4).<br>2. To assess the cost effectiveness of MAT compared to PKT from an NHS and Personal, Social Service (PSS) perspective measured using health utility, occupational status, sports participant, mental wellbeing, further surgery (treatment switching or secondary knee surgery), satisfaction with the outcome of treatment, participant global impression of change and adverse events at three (EQ-5D 5L only), 6, 12, 18 and 24 months after randomisation
Key Secondary Outcomes	KOOS4, EQ-5D-5L, Short Warwick-Edinburgh Mental Wellbeing Scale, Tegner activity/sport scale, health resource use and analgesia use at baseline, pre-intervention, 6, 12, 18 and 24 months post randomisation.<br>International Knee Documentation Committee subjective score (IKDC) at baseline and 24 months post randomisation.<br>Satisfaction with the outcome of treatment, patient global impression of change, adverse events, further knee surgery at 6, 12, 18 and 24 months post randomisation.
Ethics Review	London – Bloomsbury Research Ethics Committee, August 2022.

For peer review only

Supplementary File 3. METEOR2 SPIRIT outcomes and assessment schedule

**Time point**	**-1**	**1**		**2**		**3**	**4**	**5**	**6**	**7**
Study period	Screening	Baseline	Pre-intervention	Intervention	Medical notes review	3-month	6-month	12-month	18-month	24-month
Check eligibility	✓									
Invitation to study	✓									
Informed consent		✓								
Medical history		✓								
Inclusion/exclusion criteria		✓								
Randomisation		✓								
Intervention (Surgery/PKT)				✓						
Operation note/Physio CRF				✓						
KOOS4		✓	✓				✓	✓	✓	✓
IKDC		✓	✓							✓
EQ-5D-5L		✓	✓			✓	✓	✓	✓	✓
Health resource use			✓			✓	✓	✓	✓	✓
SWEMWS		✓	✓				✓	✓	✓	✓
Tegner Activity Scale		✓	✓				✓	✓	✓	✓
Patient global impression of change							✓	✓	✓	✓
Complications, AEs, further surgery					✓	✓	✓	✓	✓	✓
Days to initiation of treatment				✓		✓	✓	✓	✓	
Rehabilitation attendance					✓	✓	✓	✓	✓	✓

For peer review only

Supplementary File 3: TIDieR Checklist for METEOR2 PKT Intervention

		Where located	
**Item number**	**Item**	**Primary paper (page number)**	**Other (details)**
**1**	**BRIEF NAME**<br>Provide the name or a phrase that describes the intervention	5, 14-15	N/A
**2**	**WHY**<br>Describe any rationale, theory, or goal of the elements essential to the intervention	14-15	N/A
**3**	**WHAT**<br>Materials: describe any physical or informational materials used in the intervention, including those provided to participants or used in intervention delivery or in training providers.	14-15	N/A
**4**	Procedures: Describe each of the procedures, activities, and/or processes used in the intervention including any enabling or support activities.	14-15	N/A
**5**	**WHO PROVIDED**<br>For each category of intervention provider (e.g. psychologist, nursing assistant), describe their expertise, background and any specific training given.	14-15	N/A
**6**	**HOW**<br>Describe the modes of delivery (e.g. face-to-face or by some other mechanism, such as internet or telephone) of the intervention and whether it was provided individually or in a group.	14-15	N/A
**7**	**WHERE**<br>Describe the type(s) of location(s) where the intervention occurred, including any necessary infrastructure or relevant features.	14-15	N/A
**8**	**WHEN AND HOW MUCH**<br>Describe the number of times the intervention was delivered and over what period of time including the number of sessions, their schedule, and their duration, intensity or dose.	14-15	N/A
**9**	**TAILORING**<br>If the intervention was planned to be personalised, titrated or adapted, then describe what, why when, and how.	14-15	N/A
**10**	**MODIFICATIONS**<br>If the intervention was modified during the course of the study, describe the changes (what, why, when, and how)	N/A	N/A
**11**	**HOW WELL**<br>Planned: If intervention adherence or fidelity was assessed, describe how and by whom, and if any strategies were used to maintain or improve fidelity, describe them.	9, 14-15	N/A
**12**	Actual: If intervention adherence or fidelity was assessed, describe the extent to which the intervention was delivered as planned.	N/A	N/A

For peer review only

METEOR2 SPIRIT 2013 checklist

Section/item	Item No	Description	Page Number
**Administrative information**			
Title	1	Descriptive title identifying the study design, population, interventions, and, if applicable, trial acronym	1
Trial registration	2a	Trial identifier and registry name. If not yet registered, name of intended registry	3
	2b	All items from the World Health Organization Trial Registration Data Set	Supp. File 2
Protocol version	3	Date and version identifier	19
Funding	4	Sources and types of financial, material, and other support	24
Roles and responsibilities	5a	Names, affiliations, and roles of protocol contributors	1, 20
	5b	Name and contact information for the trial sponsor	24
	5c	Role of study sponsor and funders, if any, in study design; collection, management, analysis, and interpretation of data; writing of the report; and the decision to submit the report for publication, including whether they will have ultimate authority over any of these activities	24
	5d	Composition, roles, and responsibilities of the coordinating centre, steering committee, endpoint adjudication committee, data management team, and other individuals or groups overseeing the trial, if applicable (see Item 21a for data monitoring committee)	24
**Introduction**			
Background and rationale	6a	Description of research question and justification for undertaking the trial, including summary of relevant studies (published and unpublished) examining benefits and harms for each intervention	4-5
	6b	Explanation for choice of comparators	4-5
Objectives	7	Specific objectives or hypotheses	6, 8

For peer review only

Trial design	8	Description of trial design including type of trial (e.g., parallel group, crossover, factorial, single group), allocation ratio, and framework (e.g., superiority, equivalence, noninferiority, exploratory)	6
**Methods: Participants, interventions, and outcomes**			
Study setting	9	Description of study settings (e.g., community clinic, academic hospital) and list of countries where data will be collected. Reference to where list of study sites can be obtained	12
Eligibility criteria	10	Inclusion and exclusion criteria for participants. If applicable, eligibility criteria for study centres and individuals who will perform the interventions (e.g., surgeons, psychotherapists)	11
Interventions	11a	Interventions for each group with sufficient detail to allow replication, including how and when they will be administered	13-15 & Supp. File 3
	11b	Criteria for discontinuing or modifying allocated interventions for a given trial participant (e.g., drug dose change in response to harms, participant request, or improving/worsening disease)	13-15 & Supp. File 3
	11c	Strategies to improve adherence to intervention protocols, and any procedures for monitoring adherence (e.g., drug tablet return, laboratory tests)	13-15 & Supp. File 3
	11d	Relevant concomitant care and interventions that are permitted or prohibited during the trial	13-15 & Supp. File 3
Outcomes	12	Primary, secondary, and other outcomes, including the specific measurement variable (e.g., systolic blood pressure), analysis metric (e.g., change from baseline, final value, time to event), method of aggregation (e.g., median, proportion), and time point for each outcome. Explanation of the clinical relevance of chosen efficacy and harm outcomes is strongly recommended	8-10
Participant timeline	13	Time schedule of enrolment, interventions (including any run-ins and washouts), assessments, and visits for participants. A schematic diagram is highly recommended (see Figure)	7

Sample size	14	Estimated number of participants needed to achieve study objectives and how it was determined, including clinical and statistical assumptions supporting any sample size calculations	16-17
Recruitment	15	Strategies for achieving adequate participant enrolment to reach target sample size	12

Methods: Assignment of interventions (for controlled trials)

Allocation:

Sequence generation	16a	Method of generating the allocation sequence (e.g., computer-generated random numbers), and list of any factors for stratification. To reduce predictability of a random sequence, details of any planned restriction (e.g., blocking) should be provided in a separate document that is unavailable to those who enrol participants or assign interventions	12
Allocation concealme nt mechanism	16b	Mechanism of implementing the allocation sequence (e.g., central telephone; sequentially numbered, opaque, sealed envelopes), describing any steps to conceal the sequence until interventions are assigned	12
Implementa tion	16c	Who will generate the allocation sequence, who will enrol participants, and who will assign participants to interventions	12
Blinding (masking)	17a	Who will be blinded after assignment to interventions (e.g., trial participants, care providers, outcome assessors, data analysts), and how	N/A
	17b	If blinded, circumstances under which unblinding is permissible, and procedure for revealing a participant's allocated intervention during the trial	N/A

Methods: Data collection, management, and analysis

Data collection methods	18a	Plans for assessment and collection of outcome, baseline, and other trial data, including any related processes to promote data quality (e.g., duplicate measurements, training of assessors) and a description of study instruments (e.g., questionnaires, laboratory tests) along with their reliability and validity, if known. Reference to where data collection forms can be found, if not in the protocol	8-9, 17

	18b	Plans to promote participant retention and complete follow-up, including list of any outcome data to be collected for participants who discontinue or deviate from intervention protocols	8-9,17
Data management	19	Plans for data entry, coding, security, and storage, including any related processes to promote data quality (e.g., double data entry; range checks for data values). Reference to where details of data management procedures can be found, if not in the protocol	17
Statistical methods	20a	Statistical methods for analysing primary and secondary outcomes. Reference to where other details of the statistical analysis plan can be found, if not in the protocol	16-18
	20b	Methods for any additional analyses (e.g., subgroup and adjusted analyses)	16-18
	20c	Definition of analysis population relating to protocol non-adherence (e.g., as randomised analysis), and any statistical methods to handle missing data (e.g., multiple imputation)	16-18
**Methods: Monitoring**			
Data monitoring	21a	Composition of data monitoring committee (DMC); summary of its role and reporting structure; statement of whether it is independent from the sponsor and competing interests; and reference to where further details about its charter can be found, if not in the protocol. Alternatively, an explanation of why a DMC is not needed	18
	21b	Description of any interim analyses and stopping guidelines, including who will have access to these interim results and make the final decision to terminate the trial	18
Harms	22	Plans for collecting, assessing, reporting, and managing solicited and spontaneously reported adverse events and other unintended effects of trial interventions or trial conduct	15-16
Auditing	23	Frequency and procedures for auditing trial conduct, if any, and whether the process will be independent from investigators and the sponsor	18
**Ethics and dissemination**			

Research ethics approval	24	Plans for seeking research ethics committee/institutional review board (REC/IRB) approval	2-3 & 18-19
Protocol amendments	25	Plans for communicating important protocol modifications (e.g., changes to eligibility criteria, outcomes, analyses) to relevant parties (e.g., investigators, REC/IRBs, trial participants, trial registries, journals, regulators)	18-19
Consent or assent	26a	Who will obtain informed consent or assent from potential trial participants or authorised surrogates, and how (see Item 32)	12
	26b	Additional consent provisions for collection and use of participant data and biological specimens in ancillary studies, if applicable	N/A
Confidentiality	27	How personal information about potential and enrolled participants will be collected, shared, and maintained in order to protect confidentiality before, during, and after the trial	17
Declaration of interests	28	Financial and other competing interests for principal investigators for the overall trial and each study site	24
Access to data	29	Statement of who will have access to the final trial dataset, and disclosure of contractual agreements that limit such access for investigators	19
Ancillary and post-trial care	30	Provisions, if any, for ancillary and post-trial care, and for compensation to those who suffer harm from trial participation	N/A
Dissemination policy	31a	Plans for investigators and sponsor to communicate trial results to participants, healthcare professionals, the public, and other relevant groups (e.g., via publication, reporting in results databases, or other data sharing arrangements), including any publication restrictions	2 & 19
	31b	Authorship eligibility guidelines and any intended use of professional writers	N/A
	31c	Plans, if any, for granting public access to the full protocol, participant-level dataset, and statistical code	19

Appendices

For peer review only

Informed consent materials	32	Model consent form and other related documentation given to participants and authorised surrogates	Supp. File 1
Biological specimens	33	Plans for collection, laboratory evaluation, and storage of biological specimens for genetic or molecular analysis in the current trial and for future use in ancillary studies, if applicable	N/A

For peer review only

For peer review only
