# Supplementary material for: Meniscal Transplant surgery or Optimised Rehabilitation full randomised trial (MeTeOR2): a study protocol
Source: BMJ Open. 2024 Jun 3;14(6):e085125. doi: 10.1136/bmjopen-2024-085125 (PMC11149128; doi:10.1136/bmjopen-2024-085125)
Supplement: Reviewer comments [file bmjopen-2024-085125.reviewer_comments.pdf]

PEER REVIEW HISTORY

BMJ Open publishes all reviews undertaken for accepted manuscripts. Reviewers are asked to complete a checklist review form (**http://bmjopen.bmj.com/site/about/resources/checklist.pdf**) and are provided with free text boxes to elaborate on their assessment. These free text comments are reproduced below.

ARTICLE DETAILS

**TITLE (PROVISIONAL)**	The Meniscal Transplant surgery or Optimised Rehabilitation full randomised trial (MeTeOR2): a study protocol
**AUTHORS**	Arnold, Susanne; Spalding, Timothy; Parsons, Helen; Beard, David; Bradley, Helen; Crisford, Peter; Ellard, David; Ferreira, Manuela; Getgood, Alan; Guck, Jonathan; Haque, Aminul; Khan, Iftekhar; Mason, James; Milroy, Bryony; Myers, P; Parker, David; Price, Andrew; Smith, Amy; Smith, NA; Smith, Toby; Stewart, Kimberley; Underwood, Martin; Verdonk, Pete; Metcalfe, Andrew

This article was not externally reviewed at BMJ Open. Protocols that have been independently assessed prior to submission to BMJ Open are usually fast-tracked to publication on the grounds that further substantial changes will not be possible. This independent assessment will usually be external, independent review for both a substantial grant award from a non-commercial or government funder and ethics approval.
